# Latched detection of zeptojoule spin echoes with a kinetic inductance parametric oscillator

**DOI:** 10.1126/sciadv.adm7624

**Published:** 2024-04-05

**Authors:** Wyatt Vine, Anders Kringhøj, Mykhailo Savytskyi, Daniel Parker, Thomas Schenkel, Brett C. Johnson, Jeffrey C. McCallum, Andrea Morello, Jarryd J. Pla

**Affiliations:** ^1^ School of Electrical Engineering and Telecommunications, UNSW Sydney, Sydney, New South Wales 2052, Australia.; ^2^ Accelerator Technology and Applied Physics Division, Lawrence Berkeley National Laboratory, Berkeley, CA 94720, USA.; ^3^ School of Science, RMIT University, Melbourne, Victoria 3001, Australia.; ^4^ School of Physics, University of Melbourne, Parkville, Victoria 3010, Australia.

## Abstract

When strongly pumped at twice their resonant frequency, nonlinear resonators develop a high-amplitude intracavity field, a phenomenon known as parametric self-oscillations. The boundary over which this instability occurs can be extremely sharp and thereby presents an opportunity for realizing a detector. Here, we operate such a device based on a superconducting microwave resonator whose nonlinearity is engineered from kinetic inductance. The device indicates the absorption of low-power microwave wavepackets by transitioning to a self-oscillating state. Using calibrated pulses, we measure the detection efficiency to zeptojoule energy wavepackets. We then apply it to measurements of electron spin resonance, using an ensemble of ^209^Bi donors in silicon that are inductively coupled to the resonator. We achieve a latched readout of the spin signal with an amplitude that is five hundred times greater than the underlying spin echoes.

## Teaser

Zeptojoule energy microwave signals emitted by a spin ensemble are detected using a superconducting parametric oscillator circuit.

## INTRODUCTION

Over the past decade, quantum-limited parametric amplifiers operating at microwave frequencies have progressed from proof of principle to ubiquity within circuit quantum electrodynamics (cQED) experiments. Typically, these devices are operated in a linear regime, but several types of parametric amplifiers are explicitly nonlinear, such as the Josephson bifurcation amplifier (JBA) (*1*) and the Josephson parametric oscillator (JPO) (*2*–*4*). These devices essentially act as a microwave “click” detector, in that threshold detection is used to discriminate the presence or absence of a signal. Central to the design of JBAs and JPOs is the use of Josephson junctions, which provide the nonlinearity required for signal mixing. An alternative source of nonlinearity is the kinetic inductance intrinsic to thin films of disordered superconductors (*5*). In contrast to Josephson junction–based devices, superconducting microwave resonators engineered from high kinetic inductance materials retain high-quality factors when operated in tesla-strength magnetic fields (*6*, *7*) and at elevated temperatures (*8*). This has recently inspired the development of magnetic field-compatible resonant parametric amplifiers that operate close to the quantum noise limit (*8*–*12*), which have a range of applications including axion detection (*13*) and quantum computation with spin qubits (*14*).

Another application of these devices is the measurement of electron spin resonance (ESR). JPAs have already been used to push noise in ESR experiments to the quantum limit, where vacuum fluctuations of the electromagnetic field dictate the spin detection sensitivity (*15*, *16*). Several other recent works have applied nonlinear microwave amplifiers (*17*) and qubit-based sensors (*18*–*21*) using Josephson junctions to measurements of ESR to push detection sensitivities to record levels. Kinetic inductance parametric amplifiers (KIPAs) have also recently been used for ESR, where they have been demonstrated to have several advantages over JPAs (*12*). Because of their compatibility with moderate magnetic fields, KIPAs can serve as both the resonator for inductive detection of spin echo signals and the first-stage amplifier. This not only simplifies the measurement setup by obviating a separate quantum-limited amplifier, it also eliminates any insertion loss between the resonator and the first cryogenic amplifier.

Here, we extend previous works with KIPAs by operating one as a click detector rather than as a linear amplifier. By biasing the device near the threshold where its behavior transitions from a linear amplifier to a parametric oscillator, the onset of parametric self-oscillations (PSO) serves as a binary indicator for the absorption of microwave wavepackets. To distinguish this operating regime, which has not been previously demonstrated, we refer to the device here as a kinetic inductance parametric oscillator (KIPO), in analogy to the JPO which operates under a similar principle. In the following, we describe the concept of the detector, calibrate its sensitivity, and demonstrate its application in ESR measurements of an ensemble of bismuth (^209^Bi) donors in silicon (Si) that are directly coupled to the device.

## Results

### Device design

**Fig. 1.**
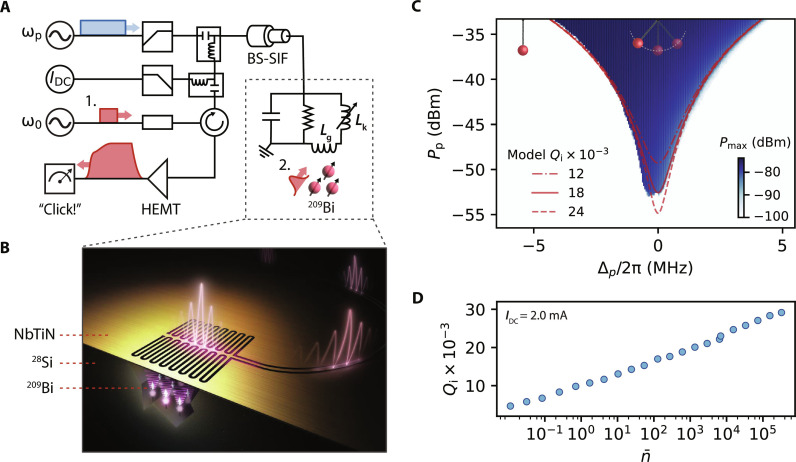
Measurement schematic and device characterization. (**A**) The device is represented by a parallel RLC resonator with frequency ω_0_ and a BS-SIF. The resonator has a geometric inductance (*L*_g_) and a kinetic inductance (*L*_k_). We operate the device as a click detector by biasing it with a strong pump with frequency ω_p_ and a DC current *I*_DC_. In this work, we detect two types of signals: weak classical signals with frequency near ω_0_ generated by a microwave source (1), which we use to calibrate the detector’s sensitivity, and spin echoes from an ensemble of ^209^Bi donor spins (2) that are resonantly coupled to the resonator via *L*_g_. Detailed schematics of the measurement setups are presented in the Supplementary Materials. (**B**) An artist’s depiction of the resonator, which is formed from a λ/4 section of transmission line with a dense interdigitated capacitor. ^209^Bi spins are implanted into the silicon substrate. (**C**) The maximum power measured with a spectrum analyzer centered at ω_0_ as a function of Δ_p_ = ω_p_ − 2ω_0_ and the pump power *P*_p_. The dark blue region corresponds to the parameter space where the device self-oscillates. The powers are referred to the output of the device, and *P*_max_ is truncated at −100 dBm to enhance clarity. The red lines correspond to the PSO threshold (*P*_th_) predicted from a model of the device using three different values of *Q*_i_. The stationary and swinging pendulums are used to depict the quiet and self-oscillating states of the device, respectively. (**D**) *Q*_i_ extracted from measurements of *S*_11_ performed with a VNA as the signal power is varied. Measurements were taken at *T* = 10 mK.

Our device is similar to the KIPAs described in previous works (*9*, *12*). It is patterned in a single lithographic step from a 50-nm-thick film of niobium titanium nitride (NbTiN) with a kinetic inductance of *L*_k_ = 3.45 pH/◻. The NbTiN is deposited on a Si substrate enriched in the isotope ^28^Si [750 parts per million (ppm) residual ^29^Si] that was implanted with ^209^Bi donors at a concentration of 10^17^ cm^−3^ over a depth of 1.35 μm. The device has a single port and consists of a quarter-wavelength (λ/4) coplanar waveguide resonator that is shorted to ground at one end and galvanically connected to a band-stop stepped impedance filter (BS-SIF) (*22*) at the other end (Fig. 1, A and B). The resonator features a dense interdigitated capacitor with 1-μm-wide fingers and a 1.5-μm gap to ground (see the Supplementary Materials), which compensates for the strong kinetic inductance and reduces the impedance of the mode to *Z*_0_ ≈ 33 ohm. The BS-SIF serves to confine the resonant mode of the device while simultaneously allowing the application of a DC bias current (*I*_DC_). An *I*_DC_ can be used to tune the resonance frequency from ω_0_(*I*_DC_ = 0)/2π = 7.776 GHz to ω_0_(*I*_DC_ = 4.89 mA)/2π = 7.530 GHz via the quadratic dependence of *L*_k_ on the total current (*23*). An *I*_DC_ also enables three-wave mixing so that a pump with frequency ω_p_ ≈ 2ω_0_ can be used to amplify signals with frequencies about ω_0_ (*9*, *24*). The device is connected to the cold finger of a dilution refrigerator with a base temperature of 10 mK or a pumped ^3^He cryostat with a base temperature of 400 mK, depending on the experiment. The DC current and microwave tones are combined at base temperature using a bias tee and diplexer (Fig. 1A). The device is measured in reflection with the signal being routed through a cryogenic high electron mobility transistor (HEMT) amplifier at 4 K. Further details of the device design and measurement setup are provided in the Supplementary Materials.

### Detector concept

The defining characteristic of PSO is the formation of a large intracavity field at ω_0_ whenever the three-wave mixing strength exceeds the average rate at which resonant photons can escape the cavity. Experimentally, this manifests as the sudden generation of a large power at ω_0_ that is emitted from the device whenever the pump power *P*_p_ is raised beyond a sharp threshold *P*_th_. In Fig. 1C, we report the maximum power recorded by a spectrum analyzer (*P*_max_) centered at ω_0_ as a function of *P*_p_ and a detuning of the pump frequency Δ_p_ = ω_p_ − 2ω_0_. The dark blue region corresponds to the parameter space where the device undergoes PSO, and the boundary of this region provides a direct measurement of *P*_th_.

Using cavity input-output theory and the Hamiltonian for the KIPA (*9*), we model our device as a parametrically driven Duffing oscillator (see the Supplementary Materials), as has been done previously for JPOs (*2*, *25*, *26*). Using parameters extracted from measurements of the device, we directly compare the model and our experimental measurement of the threshold power *P*_th_ (red lines in Fig. 1C). Crucially, the model predicts that when Δ_p_ = 0, $P_{\text{th}}\propto {\left(Q_{i}^{-1}+Q_{c}^{-1}\right)}^{2}$ , where *Q*_i_ and *Q*_c_ are the internal and coupling quality factors of the resonant mode, respectively. In Fig. 1C, we compare three models of the device which are equivalent except for *Q*_i_. For this particular measurement, we find that there is reasonable agreement between the data and model for *Q*_i_ = 18 × 10^3^. We also highlight that *P*_th_ shifts to smaller values as *Q*_i_ is increased.

The quality factors *Q*_i_ and *Q*_c_ can be extracted from measurements of the device in reflection (*S*_11_) using a vector network analyzer (VNA). As is common for superconducting microwave resonators, we observe that *Q*_i_ is nonlinear with the applied signal power, or equivalently, the average number of intracavity photons $\bar{n}$ (Fig. 1D). This indicates that two-level systems (TLSs) interact with the resonant mode (*27*) and limit *Q*_i_ at low signal power. We observe *Q*_i_ to vary between 4.7 × 10^3^ and 29 × 10^3^ for $10^{-2}<\bar{n}<3\times 10^{5}$ , which, in all cases, is much smaller than *Q*_c_ ≈ 200 × 10^3^ (see the Supplementary Materials). An important consequence of this is that the resonant mode’s linewidth $\omega_{0}\left(Q_{i}^{-1}+Q_{c}^{-1}\right)$ and hence *P*_th_ are sensitive to the signal power inside the resonator.

**Fig. 2.**
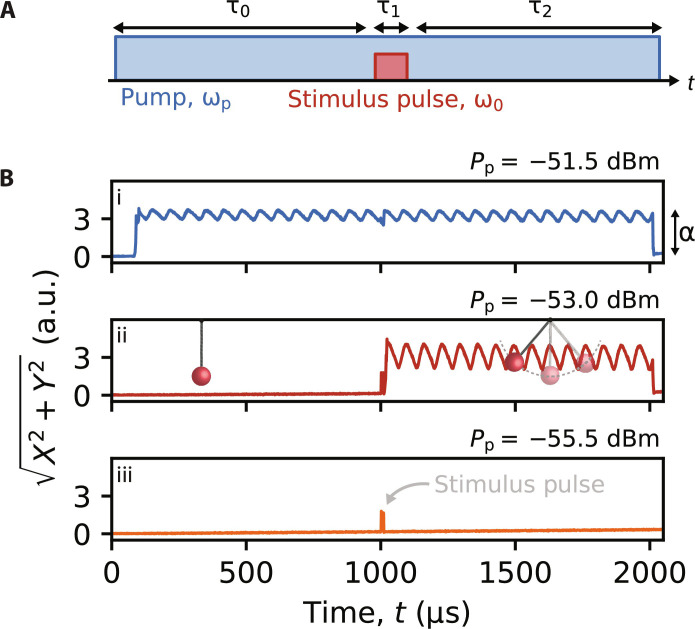
Detector concept. (**A**) A depiction of the two-tone pulse sequence. The device is biased with *I*_DC_ and pumped at ω_p_, before a short stimulus pulse with frequency ω_0_ is supplied to the device. A threshold applied to the amplitude of the demodulated signal is used to determine whether the device is self-oscillating. (**B**) Single shots of the pulse sequence for decreasing *P*_p_ (i to iii). For certain *P*_p_, the stimulus pulse triggers the onset of PSO (ii). For these experiments, τ_0_ = τ_2_ = 1 ms, τ_1_ = 10 μs, *I*_DC_ = 2.55 mA, *P*_0_ = −116.4 dBm, and *T* = 400 mK. The time is measured from the leading edge of the pump pulse. The stimulus pulse is seen directly in (iii) at *t* = 1000 μs. a.u., arbitrary units.

The experiments shown in Fig. 2 demonstrate how the dependence of *P*_th_ on $\bar{n}$ can be used to create a device that detects the absorption of low-power microwave wavepackets. First, the device is biased with a DC current *I*_DC_ = 2.55 mA and a pump tone with frequency ω_p_ = 2ω_0_. Following a delay τ_0_ = 1 ms, a stimulus pulse with duration τ_1_ = 10 μs, frequency ω_0_, and power *P*_0_ = −116.4 dBm is delivered to the input of the device (Fig. 2A). Throughout the experiment, we monitor the device by performing a homodyne measurement (i.e., demodulating the signal using a local oscillator with frequency ω_LO_ = ω_0_) and recording the amplitude of the signal quadrature components *X* and *Y*. For the lowest pump power *P*_p_ (Fig. 2B, iii), we observe no PSO. For the largest *P*_p_ (Fig. 2B, i), we observe PSO but at a time that is uncorrelated with the stimulus pulse. But for an appropriately chosen *P*_p_ ≈ *P*_th_ (Fig. 2B, ii), we observe the PSO onset at a time that is correlated with the stimulus pulse (the quiet and self-oscillating states are represented schematically with the pendulum). We hypothesize that absorption of the stimulus pulse triggers the onset of PSO due to the partial saturation of the TLSs; the increased *Q*_i_ associated with this results in *P*_th_ being dynamically reduced below the *P*_p_ setpoint, thereby triggering PSO.

From Fig. 2B, it is clear that the self-oscillating state has a large amplitude (α), relative to the stimulus pulse. Fig. 1C shows that the peak power of PSO can exceed −75 dBm. This can be understood by noting that α ∝ 1/*K* for a Duffing oscillator, where *K* is the self-Kerr strength (see the Supplementary Materials). The Kerr effect for these devices is known to be negligible relative to Josephson junction–based devices, due to the weak and distributed nature of the kinetic inductance nonlinearity (*9*). Notably, α is independent of the stimulus power, so that the onset of PSO functions as a binary indicator for the pulse absorption. By comparing the power of PSO measured with a spectrum analyzer to the average number of intracavity photons $\bar{n}$ calculated from VNA measurements of *S*_11_ with a known power, we estimate that $\bar{n}\;>10^{5}$ during PSO. For this device, α is large enough to enable subsequent four-wave mixing processes, which results in the generation of a frequency comb about ω_0_ whenever the device self-oscillates (see the Supplementary Materials). This results in oscillations in the amplitude of the demodulated signal when the device is self-oscillating (Fig. 2B).

### Detection efficiency and sensitivity

**Fig. 3.**
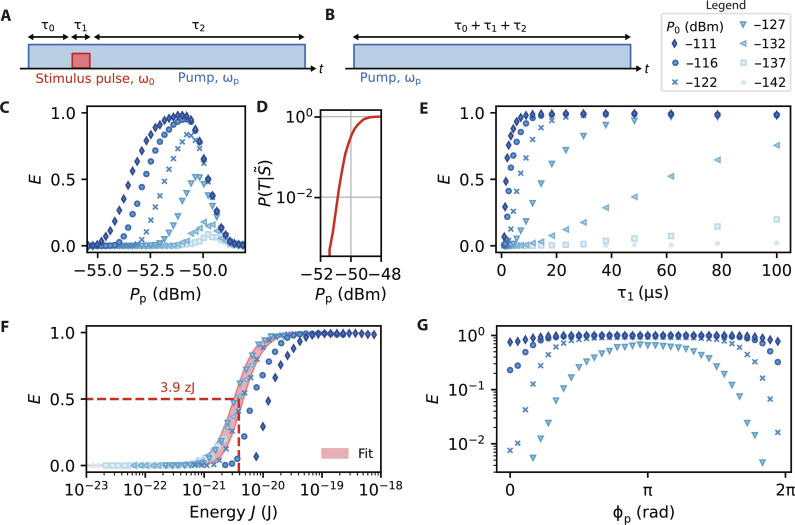
Detection efficiency measured with calibrated pulses. (**A** and **B**) The experimental pulse sequences used to measure the efficiency of the detector *E*. The sequence in (B) is a control experiment that measures dark counts. (**C**) *E* measured as a function of the stimulus pulse power *P*_0_ and pump power *P*_p_. (**D**) The probability of dark counts $P\left(T\;|\;\tilde{S}\right)$ as a function of *P*_p_. (**E**) *E* measured as a function of *P*_0_ and τ_1_ for *P*_p_ = −51.2 dBm. (**F**) The data in (E) replotted as a function of total wavepacket energy *J* = *P*_0_τ_1_. We determine the lower bound of the detector’s sensitivity by fitting the data with *P*_0_ < −116 dBm with a sigmoid function (red shaded region). (**G**) *E* measured as a function of the pump phase ϕ_p_ with τ_1_ = 10 μs. For all experiments, *I*_DC_ = 2.0 mA, τ_0_ = 10 μs, τ_2_ = 100 μs, and *T* = 10 mK. For each data point, at least 10^4^ shots of both the pulse sequences are measured. For all panels, the uncertainties in *E* are too small to be seen.

To calibrate the sensitivity of the detector, we measure its response using two pulse sequences. The first is depicted in Fig. 3A and is identical to that used in the previous section (Fig. 2A) but with different timings τ_0_, τ_1_, and τ_2_. The second sequence is similar, differing only in that the stimulus pulse is omitted (Fig. 3B). Using threshold detection, this allows us to measure the efficiency of the sensor, $E=P\left(T\;|\;S\right)-P\left(T\;|\;\tilde{S}\right)$ , where *T* indicates that the device self-oscillates and *S* ( $\tilde{S}$ ) indicates that the device receives (does not receive) a stimulus pulse. *P*(*T*∣*S*) is therefore the conditional probability describing the successful detection of the stimulus, and $P\left(T\;|\;\tilde{S}\right)$ is the probability of observing a dark count.

In Fig. 3C, we measure the detection efficiency *E* as a function of the pump power *P*_p_ with a DC current *I*_DC_ = 2.0 mA, stimulus pulse duration τ_1_ = 10 μs, and stimulus pulse powers *P*_0_ in the range [−137, −111] dBm. We note that the gain of a linear parametric amplifier (*P*_p_ < *P*_th_) operated in degenerate mode (ω_p_ = 2ω_0_) is dependent on the relative phase between the pump and the signal (*9*). For the present experiments, the relative phase between the stimulus pulse and the pump (ϕ_p_) might therefore modulate *E* and require precise calibration. To circumvent this, we phase modulate the pump microwave source at a rate of 15 kHz. While the modulation rate is slower than 1/τ_1_ = 100 kHz, for each data point, we measure 10^4^ shots of the pulse sequence to ensure the unbiased sampling of all ϕ_p_, thereby mitigating its effect. This also demonstrates that the detector can be used for phase-insensitive detection, which is useful when the phase of the signal to be detected is not known or cannot be stabilized with respect to the pump. *E* is found to grow monotonically with *P*_0_ and is nonzero over a 7-dB range in *P*_p_. For the largest *P*_0_ measured (*P*_0_ = −111 dBm), *E* reaches a maximum of 0.98, which indicates that the device detects the stimulus pulses with high probability and a small number of dark counts. As *P*_0_ is reduced, the *P*_p_ at which *E* is maximized grows slightly (from *P*_p_ = −51.2 dBm for *P*_0_ = −111 dBm to *P*_p_ = −49.4 dBm for *P*_0_ = −137 dBm). This reflects that as *P*_0_ is reduced, the device needs to be biased increasingly closer to *P*_th_ for successful detection, which results in a corresponding increase to the number of dark counts. In Fig. 3D, we show that the probability of dark counts $P\left(T\;|\;\tilde{S}\right)$ grows from <0.01 to >0.99 over a 3.1-dB range in *P*_p_. This corresponds to a dark count rate that is <0.8 Hz for *P*_p_ < −51.4 dBm and >23 kHz for *P*_p_ = −48.3 dBm (see the Supplementary Materials). We note that while we focus here on the setpoint *I*_DC_ = 2.0 mA, the device achieves high *E* for setpoints between 1.5 and 3.5 mA, corresponding to a tunable bandwidth of ∼100 MHz (see the Supplementary Materials).

Next, we measure the detection efficiency *E* as a function of the stimulus pulse duration τ_1_ (Fig. 3E). We fix the pump power *P*_p_ to the value where *E* was maximized in the previous experiment (−51.2 dBm) and continue to phase modulate the pump. In this experiment, the dark count probability $P\left(T\;|\;\tilde{S}\right)$ is at maximum 1.5 × 10^−2^, which ensures that *E* mainly reflects the probability of true detection *P*(*T*∣*S*). *E* reaches a maximum of *E* = 0.995 and is found to grow monotonically with both *P*_0_ and τ_1_. This suggests that *E* is strongly correlated with the energy of the wavepacket *J* = *P*_0_τ_1_. We confirm this by plotting *E*(*J*) (Fig. 3F), where we see that for all *P*_0_, *E*(*J*) resembles an activation curve. Fitting the entire dataset with a sigmoid, we infer that *E* = 0.5 for $J=5.0_{4.0}^{6.3}$ zJ, where the upper and lower bounds correspond to the uncertainty in the calibration of *P*_0_. We note that the *E* achieved for a given *J* does show some dependence on *P*_0_, with the two largest *P*_0_ measured having the lowest *E*. For these two measurements, the cavity ring-down time is as long as $2\omega_{0}\left(Q_{i}^{-1}+Q_{c}^{-1}\right)\approx 1.1\;\mu s$ , which reduces the detection sensitivity to pulses with short τ_1_ at such high powers. This may explain why their activation curves (Fig. 3F) do not align with those taken at lower *P*_0_, where *Q*_i_ (and therefore the ring-down time) is reduced. Excluding the data with *P*_0_ ≥ −116 dBm from the fit (red shaded area in Fig. 3F), we find the lower bound for the sensitivity to be $J=3.9_{3.1}^{4.9}$ zJ for *E* = 0.5. This corresponds to wavepackets containing $756_{601}^{952}$ microwave photons, measured at the input of the device. A complementary method for determining the sensitivity of a detector is plotting the receiver operating characteristic curve (see the Supplementary Materials), where we find that the detector performs better than a random binary classifier for pulse energies above $0.21_{0.17}^{0.27}$ zJ ( $42_{33}^{52}$ photons).

Last, we examine the influence of the pump phase ϕ_p_ on the detection efficiency *E* by turning off phase modulation on the pump microwave source and instead controlling for and stepping ϕ*_p_* throughout the experiment (Fig. 3G). For each pump power *P*_p_, we found that *E* could be both enhanced and suppressed by controlling ϕ*_p_*, relative to a control experiment where the pump microwave source was phase modulated. We also found that *E* averaged across all ϕ*_p_* agreed closely with the efficiency obtained with phase modulation. This confirms for the experiments in Fig. 3 (C to F) that although 1/τ_1_ is faster than the phase modulation rate, the large number of shots taken ensures that *E* is independent of ϕ_p_ when phase modulation is enabled. For *P*_0_ = −127 dBm (the weakest power measured in this experiment), *E* could be suppressed to zero or made as large as 0.68, while with phase modulation, *E* = 0.21. The phase dependence of *E* highlights that the microwaves absorbed into the resonator are first parametrically amplified and therefore saturate the TLSs more effectively than they would without a strong parametric pump. The sensitivity of the detector can therefore be optimized by operating it in a phase-sensitive manner.

### Latched readout of a spin ensemble

Next, we use the device to detect ESR of the ^209^Bi spin ensemble that was implanted into the Si substrate. At low temperatures, the ^209^Bi donors bind one extra valence electron compared to the surrounding Si atoms. The bound electron (*S* = 1/2) and nuclear (*I* = 9/2) spins are coupled via the hyperfine interaction *H*_A_/ℏ = *AS* · *I*, where *A*/2π = 1.475 GHz is the hyperfine strength and *S* and *I* are the electron and nuclear spin operators, respectively. At fields *B*_0_ < 100 mT, the hyperfine interaction strength is comparable to that of the Zeeman interaction *H*_B_ = *B*_0_(γ_e_*S*_z_ − γ_n_*I*_z_), where γ_e_/2π = 27.997 GHz/T and γ_n_/2π = 6.96 MHz/T are the electron and nuclear spin gyromagnetic ratios, respectively. Under these conditions, the eigenstates of the spin Hamiltonian *H* = *H*_A_ + *H*_B_ are best given as *F*, *m*_F_, where *F* = *S* + *I* is the total spin and *m*_F_ is its projection onto *B*_0_. Bismuth donors can have exceptional spin relaxation and coherence times, with *T*_1_ > 1600 s (*28*) and *T*_2_ as long as 700 ms (*29*), while the large zero-field splitting of the system 5*A*/2π = 7.375 GHz makes it ideal for applications involving superconducting devices (*30*, *31*).

**Fig. 4.**
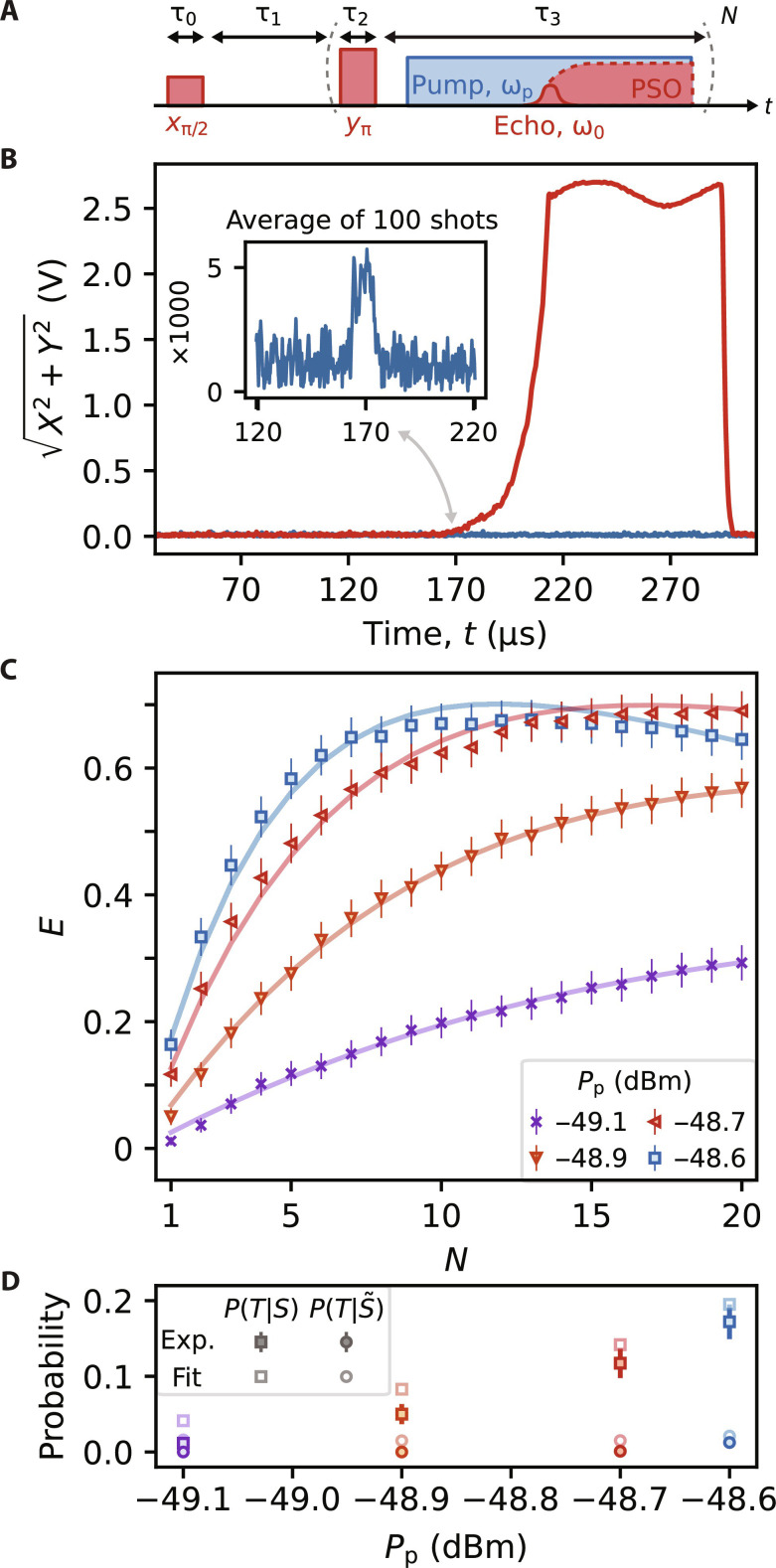
Latched readout of ^209^Bi spin echoes. (**A**) A schematic of the Hahn echo (*N* = 1) and CPMG-*N* pulse sequences. For the pulses, *x* and *y* refer to the phase of the signal while π/2 and π refer to the tipping angle. When the pump is on, the echo may trigger PSO. $P\left(T|\tilde{S}\right)$ was measured using sequences where either the *x*_π/2_ or *y*_π_ pulses were omitted, so that no spin echo was produced. Both control sequences were performed and resulted in a similar number of dark counts. (**B**) Single shots of the Hahn echo pulse sequence with the pump off (blue) and with with *P*_p_ = −48.6 dBm (red), measured at a field of *B*_0_ = 13.71 mT. Inset: An average of 100 Hahn echoes measured with the pump off—note the scaling of the *y* axis. The double-sided arrow highlights the correspondence between the time of the spin echo and the onset of PSO. (**C**) *E* measured as a function of *N*. For each *P*_p_, a total of 1040 shots of the pulse sequence with *N* = 20 were measured. The errorbars correspond to the 95% confidence interval. The solid lines are fits of the data to Eq. 1. (**D**) A comparison between the experimental values of *P*(*T*∣*S*) and $P\left(T\;|\;\tilde{S}\right)$ for *N* = 1 and those fit to the data using Eq. 1. All experiments were completed at *T* = 400 mK with *I*_DC_ = 2.55 mA and with the pump phase modulated at a rate of 15 kHz.

In our experiments, we apply an in-plane magnetic field of strength *B*_0_ = 13.71 mT to bring the ∣4, 4⟩ ↔ ∣5, 5⟩ spin transition into resonance with the device. Resonant pulses delivered to the device are then used to control the sub-ensemble of spins with Larmor frequencies within the bandwidth of the resonator. The spins are measured using a Hahn echo pulse sequence, which is depicted in Fig. 4A. The first pulse in the sequence (*x*_π/2_) is a π/2 pulse which causes the spins to precess about *B*_0_ and dephase due to interactions with their environment. After a time delay τ_1_, a phase-shifted π pulse (*y*_π_) partially reverses the dephasing and causes the spins to refocus and emit an echo, temporarily populating the resonator with photons (*32*). For the Hahn echo pulse sequence, this refocusing procedure is performed only once, which we designate *N* = 1 (see Fig. 4A).

To detect the spins via PSO, we modify the standard Hahn echo pulse sequence by adding in a strong parametric pump following the refocusing pulse. As in the previous experiments, we set the pump power *P*_p_ close to but below the threshold power *P*_th_. The spin echo then serves as the stimulus pulse and triggers PSO when it populates the resonator with microwave photons. Individual shots of the pulse sequence are depicted in Fig. 4B. When no pump tone is supplied (blue trace), the device functions simply as a resonator. The first amplifier in the measurement chain is a HEMT at 4 K. As a result, the signal-to-noise ratio (SNR) is poor, and to reliably resolve the echo, the sequence must be repeated so that the signal can be averaged (inset of Fig. 4B). When the pump is on, the echo may trigger PSO, resulting in a detection signal with an amplitude that is a factor ∼500 greater than that of the echo (red trace). Moreover, while the echo itself is ≈10 μs long, corresponding to the duration of the resonant pulses used for spin control [as often observed when performing spin resonance on highly coherent spin systems with high-quality factor resonators (*12*, *15*)], the PSO persists until the pump is turned off. This latched ESR readout of a spin ensemble constitutes an original technique for detecting spin resonance.

A common method used to enhance the speed of ESR measurements is to average multiple spin echoes collected with a Carr-Purcell-Meiboom-Gill (CPMG) sequence. The CPMG pulse sequence is an extension of the Hahn-echo pulse sequence that refocuses the spins with *y*_π_ pulses a total of *N* times (Fig. 4A). Neglecting the finite decay of the spin echo throughout the pulse sequence, the SNR of a CPMG measurement is equivalent to that of *N* Hahn echoes. Notably, however, the amount of measurement time required to achieve a fixed SNR is reduced when using CPMG, because the *N* echoes are collected within a single shot of the pulse sequence rather than having to wait for the longitudinal magnetization of the spins to recover (in a time *T*_1_) before collecting each echo. In Fig. 4C, we show that this technique can similarly be applied to boost the detection efficiency *E* of our detector and thereby reduce the amount of measurement time required to detect a spin signal. For each *P*_p_, we perform 1040 shots of the pulse sequence with *N* = 20 refocusing pulses. Because the spins are directly coupled the detector, they are driven by the PSO whenever they are triggered, such that we do not anticipate additional echoes after a detection event. Therefore, we calculate *E*(*N*) by determining whether PSO were triggered for any of the *N* repetitions within each shot of the pulse sequence. We find that by repeatedly refocusing the spin echo, *E* can be increased by up to a factor of 25 for *P*_p_ = −49.1 dBm, which, in absolute terms, corresponds to an improvement in *E* by 0.28. For the three larger values of *P*_p_ measured, the absolute improvement to *E* is even greater, with all being enhanced by more than 0.5 for a modest number of repetitions *N*.

The CPMG-*N* experiments can be modeled in a simplified way by treating the detection of each of the *N* echoes as statistically independent events, with constant probabilities for spin echo detection *P*(*T*∣*S*) and dark counts $P\left(T\;|\;\tilde{S}\right)$ . The model therefore assumes that the amplitude of the spin echo does not decay appreciably within the time required to complete the *N* refocusing pulses. After *N* echoes, the probability of detecting a spin echo is 1 − [1 − *P*(*T*∣*S*)]*^N^*, and the probability of a dark count is $1-{\left[1-P\left(T\;|\;\tilde{S}\right)\right]}^{N}$ . The efficiency *E*(*N*) is their difference and is given by

$$E\left(N\right)={\left[1-P\left(T\;|\;\tilde{S}\right)\right]}^{N}-{\left[1-P\left(T\;|\;S\right)\right]}^{N}$$ (1)

In Fig. 4C, we show that the experimental measurements of *E*(*N*) are well-fit by Eq. 1. We also compare the values of the spin echo detection probability *P*(*T*∣*S*) and the dark count probability $P\left(T\;|\;\tilde{S}\right)$ fit to the data with those measured using a Hahn echo sequence (*N* = 1) (Fig. 4D). The general agreement between the two sets of values confirms that the approximation is appropriate for this experiment.

An important consequence of Eq. 1 is that for a CPMG experiment there is a maximum efficiency *E*(*N*) and therefore an optimal number of refocusing pulses *N*. Moreover, this optimal *N* scales favorably as $O\{\text{ln}\left[P\left(T\;|\;S\right)/P\left(T\;|\;\tilde{S}\right)\right]\}$ (see the Supplementary Materials). We see in Fig. 4C that for *P*_p_ = −48.6 dBm where $P\left(T\;|\;S\right)/P\left(T\;|\;\tilde{S}\right)=5.3$ , *E* could be maximized with only *N* = 13 refocusing pulses. This suggests that the CPMG detection scheme should also be effective on spin systems with shorter coherence times, as are commonly found in conventional ESR spectroscopy experiments (*33*), where the number of refocusing pulses that can be applied is limited. Measuring spins with lower coherence would likely require an increase in both the excitation and detection bandwidth relative to the present device.

## DISCUSSION

The sensitivity of the detector could be improved beyond what is demonstrated here by designing a device that is critically coupled (*Q*_i_ = *Q*_c_), in which case the wavepacket would be more efficiently absorbed by the device. For the current detector, *Q*_c_/*Q*_i_ ≈ 24 for $\bar{n}\approx 1$ , in which case the fraction of power that is absorbed is only 1 − ∣*S*_11_∣^2^ ≈ 0.15. Reducing *Q*_c_ to achieve critical coupling, e.g., by modifying the BS-SIF, could therefore increase its sensitivity by nearly an order of magnitude while simultaneously increasing the bandwidth. We also note that the peak sensitivity was measured in an experiment using phase modulation of the pump microwave source, while in Fig. 3G, we have already demonstrated that *E* can be increased by more than a factor of three, provided the pump and signal are phase-matched. There may be opportunities to improve the phase-insensitive detection efficiency through optimizing aspects of the pump phase modulation or by considering alternative strategies, such as intentionally detuning the pump frequency.

The sensitivity of future devices could be further enhanced by increasing the strength with which the device couples to TLSs. While this approach is counter to most cQED experiments, in the present case, it will increase the sensitivity of *Q*_i_ to the average number of intracavity photons $\bar{n}$ , which is central to signal detection. This could be achieved by reducing the widths of the coplanar waveguide gap (*g*) and conductor (*w*) below the dimensions of the present device (*g* = 1.5 μm, *w* = 1 μm). Because TLSs reside at the dielectric interface, this will have the effect of concentrating the resonant mode volume in the material hosting the TLSs (*27*, *34*). In addition, dielectrics with higher concentrations of TLSs (*35*–*37*) could be intentionally deposited to improve both the detection sensitivity and bandwidth. If the sensitivity can be improved to the single photon level, the KIPO could be used as a detector of itinerant microwave photons, which might make it applicable in schemes for generating remote entanglement between qubits (*38*) or nonclassical states of light (*39*).

The experiments presented in Fig. 4 are a proof of concept that demonstrate how the detector might be used in ESR experiments. While the experiments in Fig. 4 are taken at a single magnetic field *B*_0_, we also compare the conventional ESR and KIPO methods over a range of *B*_0_ fields, simulating how the KIPO might be used as a spectroscopic probe (see the Supplementary Materials). This experiment further highlights that while detection of the spin echoes with the KIPO occurs with probability <1, the KIPO signal is greater than conventional ESR methods even when averaging over all shots and accounting for dark counts. This is directly related to the large power of the PSO signal, which we show in Fig. 1C can exceed −75 dBm at its peak, referred to the output of the device. This is ∼20 dB greater than the equivalent room temperature Johnson-Nyquist noise measured over a bandwidth of 100 MHz, which means that it would be possible to measure “clicks” produced by the KIPO even without a cryogenic HEMT amplifier.

The experiments shown in Fig. 4, (C and D) show that ESR detection using the KIPO benefits from the extension of conventional ESR techniques. Further, it was recently demonstrated how click detectors can be used to perform a full suite of ESR techniques (*19*), which may be similarly adapted to the KIPO. Continuous-wave ESR detection might also be explored, as demonstrated using classical oscillator circuits that operate deep in the oscillator regime (*40*). In comparing the KIPO to works using Josephson junction–based devices, we emphasize the simplicity of our experiment. The KIPO does not require any qubits for detecting the microwave signals and can be operated at elevated temperatures and directly in a magnetic field (here 400 mK and 13.71 mT, which would preclude the use of aluminum Josephson junctions). Ultimately, this enables the KIPO to be coupled directly to the spin ensemble, which obviates a following quantum-limited amplifier or a cryogenic HEMT amplifier, as noted above. Future work will focus on comparing both detection techniques and further exploring the advantages of latched echo readout in ESR spectroscopy.

## MATERIALS AND METHODS

### Device fabrication

The silicon substrate is enriched in the isotope ^28^Si with a residual ^29^Si concentration of 750 ppm. ^209^Bi ions with multiple energies were implanted to a concentration of 10^17^ cm^−3^ between 0.75- and 1.75-μm depth and electrically activated with a 20-min anneal at 800°C in a N _2_ environment. A 50-nm film of NbTiN was then sputtered (STAR Cryoelectronics). The film was determined to have a kinetic inductance of 3.45 pH/◻ by matching the resonant frequencies of capacitively coupled λ/4 resonators to microwave simulations (Sonnet). The device was patterned in a single step with electron beam lithography and subsequently dry-etched with a CF_4_/Ar plasma. The device was then mounted and wire bonded to a printed circuit board and enclosed in a three-dimensional copper cavity.

### Measurement setups

The measurements are performed in two different cryogenic systems: a dry dilution refrigerator with a base temperature of 10 mK and a pumped ^3^He cryostat with a base temperature of 400 mK. In both setups, DC and microwave signals are combined at the coldest stage with a bias-tee and diplexer, and two cryogenic circulators are used to route the reflected microwave signals to cryogenic HEMT amplifiers. For the ESR measurements performed in the ^3^He refrigerator, a solenoid is used to generate the *B*_0_ field. The cryogenic circulators are placed outside of the magnet bore and contain two layers of magnetic shielding to protect them from any stray magnetic fields. In some experiments, additional amplification and filtering of the microwave and baseband signals are performed at room temperature. Time resolved measurements are performed via homodyne detection with ω_LO_ = ω_p_/2 using a homemade microwave bridge. The Supplementary Materials includes detailed schematics of both setups.

### VNA measurements

VNA measurements were fit to cavity input-output theory (*41*) to extract ω_0_, *Q*_i_, and *Q*_c_. For the data in Fig. 1D, a baseline was measured and subtracted from each measurement, by setting *I*_DC_ = 0 mA, which shifted ω_0_ outside the measurement window.

### Measurement of *E*

The measurements of *E* presented in Fig. 3 were collected by repeating each pulse sequence 500 shots at a time, with a 33-Hz repetition rate (pump duty cycle ≈1/250). In each experiment, the parameters being swept (*P*_0_, *P*_p_, τ_1_, ϕ_p_, and the corresponding control experiments) were selected in pseudo-random order. For each datapoint, a minimum of 10^4^ shots of both the experimental and control sequences were run. A threshold applied to the amplitude of the demodulated signal $\sqrt{X^{2}+Y^{2}}$ was used to determine whether the device self-oscillated. For the experiments in Fig. 3 (A to D), the pump was phase-modulated at a rate of 15 kHz to ensure that *E* would not be dependent on ϕ_p_. In Fig. 3E, phase modulation was disabled. Despite a 3-GHz clock used to sync the two microwave sources, a small phase drift was still evident in *P*(*T*∣*S*) [the control experiments used for calculating $P\left(T\;|\;\tilde{S}\right)$ showed no trend, as expected]. To correct for the phase drift, *P*(*T*∣*S*) for each experimental repetition (500 shots for each ϕ_p_) was fit with a phenomenological function and aligned with the minimum *E* on ϕ_p_ = 0. For all measurements of *E*, the uncertainties were found by adding in quadrature the 95% confidence intervals for the binomnial distributions of *P*(*T*∣*S*) and $P\left(T\;|\;\tilde{S}\right)$.

### Measurements of ^209^Bi

All measurements of ^209^Bi were performed at 400 mK. The device is mounted so that *B*_0_ is aligned parallel to the long axis of the resonator. This orientation is chosen so that the magnetic field of the resonant mode *B*_1_ is perpendicular to *B*_0_ for spins located underneath the resonator. Numerically solving the ^209^Bi spin Hamiltonian allows one to target specific ^209^Bi ESR transitions by adjusting *B*_0_ until the spins are resonant with the cavity. In this work, we measure the ∣4, 4⟩ ↔ ∣5, 5⟩ spin transition. The small discrepancy between the *B*_0_ at which we measure ESR (13.71 mT) and the numerically predicted field (13.54 mT) might be attributed to strain caused by the different coefficients of thermal expansion of the Si substrate and NbTiN thin film (*42*) or a slight miscalibration of the superconducting solenoid used to generate *B*_0_. For the Hahn echo and CMPG sequences used for the experiments in Fig. 4, we use τ_0_ = τ_2_ = 10 μs, τ_1_ = 150 μs, and τ_3_ = 2τ_1_ + 4τ_0_/π (*43*). The leading edge and trailing edges of the pump pulses are padded by 50 and 30 μs with respect to the π*_y_* pulses, to avoid their amplification. The sequences were measured with a repetition time of 7 s. This is similar in magnitude to the spin relaxation rate *T*_1_ ≈ 12 s, which, in our experiments, is limited by spin diffusion (*12*). For the experiment in Fig. 4C, a total of 1040 shots of the full sequence with *N* = 20 were measured for both the control and experimental sequences at each *P*_p_ while alternating between the control and experimental sequences. We performed two independent control sequences: one where the *x*_π/2_ pulse was excluded and a second where the twenty *y*_π_ pulses were excluded. The two values of $P\left(T\;|\;\tilde{S}\right)$ were found to agree closely with one another, despite the different timing of the pulses relative to the onset of the pump. This indicates that the dark count rate was not influenced by residual fields of the *x*_π/2_ and *y*_π_ pulses in this experiment. Histograms of the detector counts for the experimental and control sequences as a function of time and repetition *N* are provided in the Supplementary Materials.

### Calibration of powers

All powers mentioned throughout the text are referred to the input of the device enclosure. To calibrate these powers, we performed three separate cooldowns of the cryostats with two additional high-frequency lines (*L*_1_ and *L*_2_). In the first two cooldowns, *L*_1_ or *L*_2_ was connected in place of the device, and *S*_21_ measurements of each pair of lines were taken. In the third cooldown, an *S*_21_ measurement was taken where *L*_1_ and *L*_2_ were connected to one another at the base temperature plate. The combination of *S*_21_ measurements taken over the three cooldowns allowed for a full reconstruction of the gain (loss) of each line, which agreed closely with the designed amplification (attenuation). We estimate the powers to be accurate to within ±1 dB.

## Supplementary Materials

### This PDF file includes:

Supplementary text Figs. S1 to S14 Table S1 References
