# Supplementary material for: Latched detection of zeptojoule spin echoes with a kinetic inductance parametric oscillator
Source: Sci Adv. 2024 Apr 5;10(14):eadm7624. doi: 10.1126/sciadv.adm7624 (PMC10997192; doi:10.1126/sciadv.adm7624)
Supplement: Supplementary file 1 — Supplementary text Figs. S1 to S14 Table S1 References [file sciadv.adm7624_sm.pdf]

Science Advances
AAAS

Supplementary Materials for

Latched detection of zeptojoule spin echoes with a kinetic inductance parametric oscillator

Wyatt Vine *et al.*

Corresponding author: Jarryd J. Pla, jarryd@unsw.edu.au

Sci. Adv. **10**, eadm7624 (2024)
DOI: 10.1126/sciadv.adm7624

This PDF file includes:

Supplementary text
Figs. S1 to S14
Table S1
References

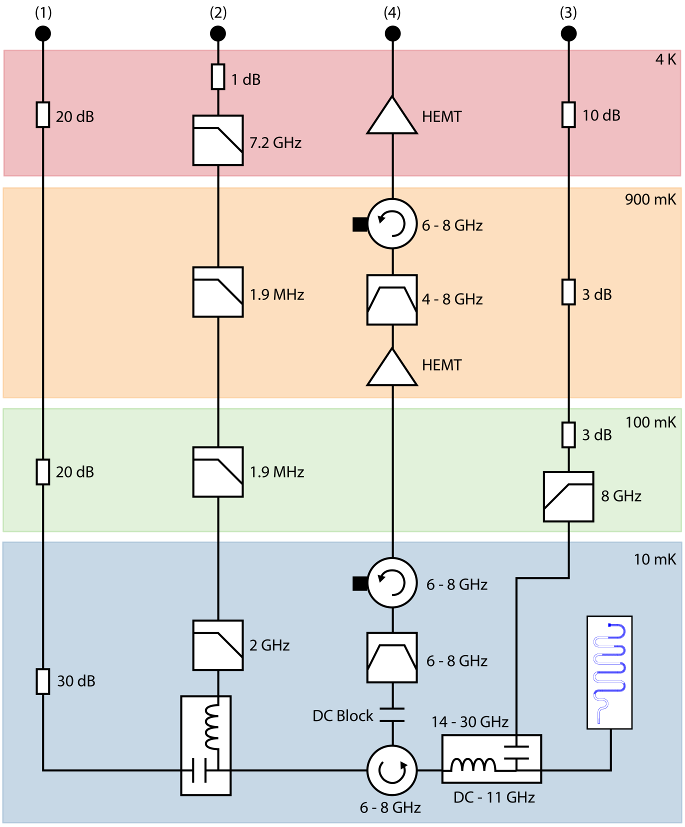

FIG. S1. Schematic for measurements performed at 10 mK.

I. MEASUREMENT SETUPS

A. Measurements at 10 mK

Measurements at 10 mK were performed using a Bluefors LD-250 dilution refrigerator. The copper sample enclosure was mounted directly to the mixing chamber (MXC) plate. We utilized 4 lines in the experiment, labeled (1)-(4) in Fig. S1. (1) The signal line was used to supply tones near the resonance frequency of the device ($\omega_0/2\pi \approx 7.7$ GHz). (2) The DC line was used to apply a DC-current to the device. (3) The pump line was used to send tones near twice the resonance frequency of the device ($\omega_\mathrm{p}/2\pi \approx 15.4$ GHz). (4) The output line was used to amplify the reflected microwave signals. The signals on lines (1), (2) and (3) were combined with a bias-tee (Pasternack PE1615) and a diplexer (Marki DPX114) and fed into the device via a short semi-rigid cable connected to the common port of the diplexer.

To ensure that the noise reaching the device at ω_0 was limited by the equilibrium fluctuations at the lowest temperature stage (10 mK), 70 dB of fixed broadband attenuation was used on line (1) and a series of reflective low-pass filters were used on line (2). To facilitate the application of a strong pump, line (3) had only 16 dB of fixed attenuation, but high-pass filters at the lowest temperature stages ensured > 60 dB of attenuation for frequencies about ω_0.

The reflected microwave signals were routed through two cryogenic HEMT amplifiers situated at 1 K and 4 K. To protect the amplifiers from damage and radiation from reaching the sample, a DC-block, two band-pass filters, and two isolators were used.

B. Measurements at 400 mK

Measurements at 400 mK were performed using a ^{3}He refrigerator. The copper sample enclosure was mounted to the base-plate of the insert and enclosed in a vacuum can that was dipped directly

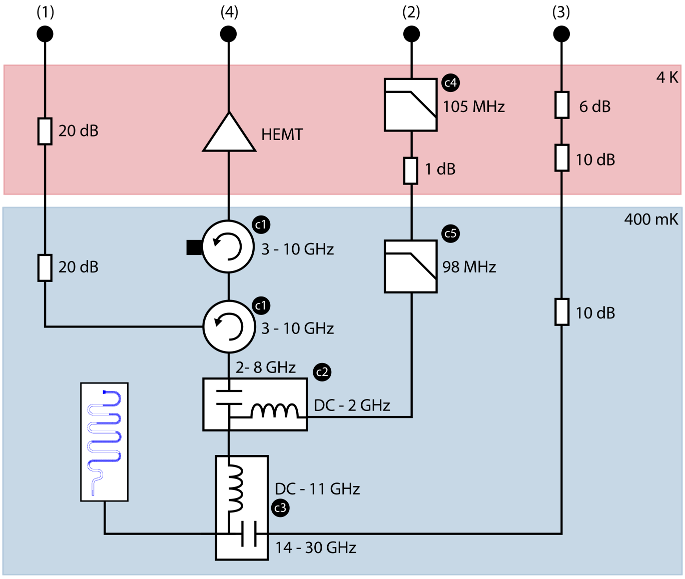

FIG. S2. Schematic for measurements performed at 400 mK. The components are (c1) Raditek RADC-4-10-Cryo circulator, (c2) Marki DPXN4, (c3) Marki DPX1114, (c4) Mini-Circuits VLF-105+, (c5) Mini-Circuits SLP-100+.

into liquid ^{4}He. A closed-cycle, single-shot ^{3}He system maintained the sample at $\sim$ 400 mK throughout the duration of the measurements. The attenuation and filtering of the four lines was configured similarly to that of the 10 mK measurements.

C. Microwave Bridge

Time-resolved experiments were performed using a homemade microwave spectrometer which is depicted in Fig. S3. The system has two main paths: one for generating phase-coherent pulses and one for performing homodyne demodulation of the signals coming from the device. Pulses are generated by IQ mixing a local oscillator (LO) with baseband signals from an arbitrary waveform generator (AWG). To extend the dynamic range of the system, a programmable attenuator is used to vary the output power of the system. To suppress leakage of signals from the box when no pulses are being sent, a fast microwave switch is placed before the output of the system and is actuated throughout the pulse sequences. Similar switches are placed at the input of the box and provide $>$ 40 dB of attenuation when open to ensure that the high power pulses used for performing ESR do not reach the demodulator with a power exceeding its damage threshold. Power entering the box is further amplified before being homodyne demodulated. The resulting quadrature signals, $X(t)$ and $Y(t)$, are digitized by a data acquisition system (DAQ). For experiments where the pump is added to a pulse sequence, it is routed through the box and gated by a fast microwave switch. Triggering of the AWG, DAQ, and microwave switches is achieved with TTL logic supplied by a pulse generator (Spin-Core PulseBlaster ESR Pro).

II. DEVICE DESIGN

The design of the device is similar to that of several previously reported Kinetic Inductance Parametric Amplifiers (KIPAs) [7, 10]. It consists of two main components: a high-quality factor quarter-wavelength ($\lambda/4$) microwave resonator and a Band-Stop Stepped Impedance Filter (BS-SIF, Fig. S4). The former is constructed as a dense Interdigitated Capacitor (IDC) with forty fingers of width 1 μm extending from the center conductor, each of which has a spacing of 1.5 μm to ground (Fig. S4b). The fingers of the IDC were made to have two lengths (twenty with length 125 μm and twenty with length 135 μm) to produce a frequency detuning of the next harmonic

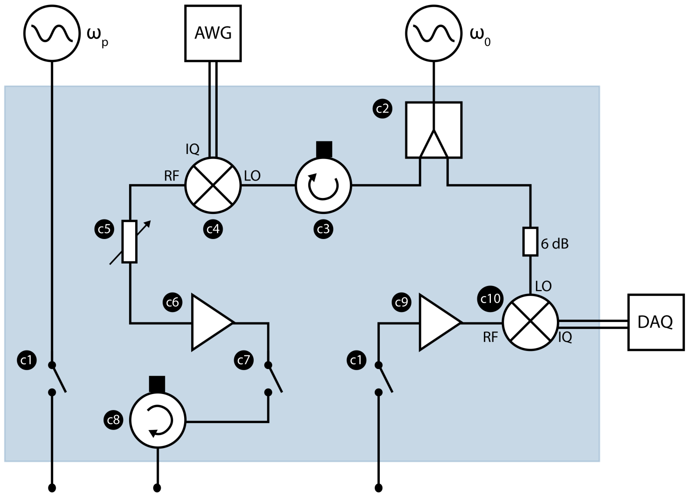

FIG. S3. Schematic for the home-built spectrometer used for pulsed ESR measurements. c1: single-pole double-throw microwave switch, c2: Marki PD-0R510, c3: Ditom D3C4080, c4: Marki IQ-4509LXP, c5: Mini-Circuits RUDAT-13G-60, c6: Mini-Circuits ZVE 3W-183+, c7: double-pole quad-throw microwave switch, c9: Mini-Circuits ZX60-05113LN+, c10: Polyphase AD60100B, ω_0 microwave source: Keysight PNA-L N5231B, ω_p microwave source: Anritsu MG3692b, AWG: Keysight M3202A, DAQ: Keysight M3102A. Triggering of the AWG, DAQ, and microwave switches is achieved with TTL logic supplied by a pulse generator (Spin-Core PulseBlaster ESR Pro).

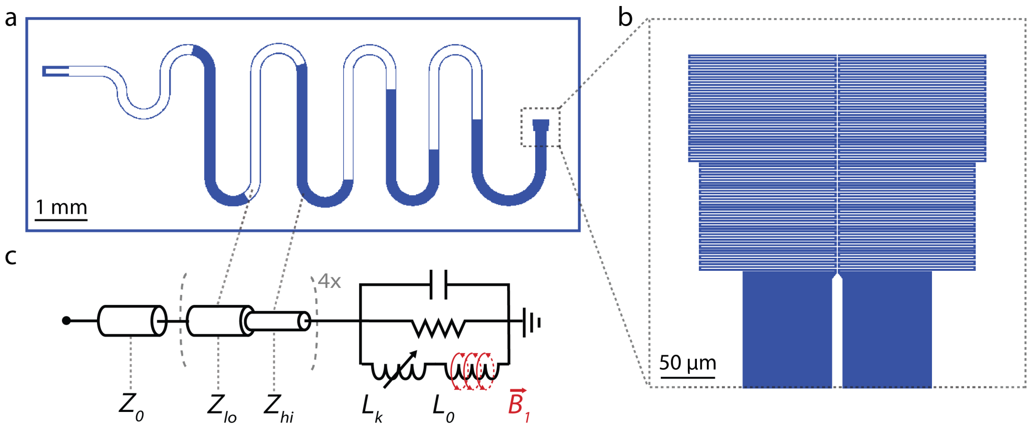

FIG. S4. Device schematic. (a) A top-down view of the full device. The white regions correspond to NbTiN and the blue regions where it has been etched to reveal the silicon underneath. The large meandering CPW corresponds to the BS-SIF (note that at this scale the centre conductor of the Z_hi section is too small to be seen). (b) A close-up view of the $\lambda/4$ IDC resonator. It is galvanically connected to the BS-SIF and shorted to ground. The IDC has a total of forty fingers extending from the centre conductor of the CPW, with two different lengths. (c) A lumped-element circuit model of the device. The resonator has two contributions to its total inductance: a kinetic inductance L_k and a geometric inductance L_0. The former is non-linear and responsible for three-wave mixing, while the latter gives rise to an AC magnetic field B_1 that couples to ^{209}Bi donors implanted into the silicon substrate.

at ω_1. This ensures $\omega_1 \neq 3\omega_0$, so that a strong pump tone at $\omega_\mathrm{p} = 2\omega_0$ does not couple the $\lambda/4$ and $3\lambda/4$ modes. The BS-SIF consists of eight total segments of CPW with alternating low $Z_\mathrm{lo} = 29.5$ Ohms and high $Z_\mathrm{hi} = 123$ Ohms impedance. The electrical lengths of each segment are $\lambda/4$ at the resonant frequency ω_0, which results in a deep stop-band centered at ω_0, and pass-bands at DC and ω_p. The BS-SIF therefore serves two purposes: it isolates the resonant mode from the measurement port (i.e. creates a large coupling quality factor Q_c) and it enables the resonator to be galvanically connected to the measurement port so that it can be biased with a DC current I_DC. The design parameters of the device are summarized in Table S1.

Parameter	$Z_{\rm hi}$ (BS-SIF)	$Z_{\rm lo}$ (BS-SIF)	Resonator Centre Conductor	Resonator Fingers
W (μm)	10	162	1	1
G (μm)	82	6	2	1.5
Length (μm)	3218	3388	201	120, 130
Z (Ohms)	123	29.5	-	-

TABLE S1. KIPA design parameters. W and G are the width of the CPW conductor and gap, respectively. The impedance Z of the BS-SIF segments is found through simulations in the software package Sonnet. The impedance of the IDC resonator is estimated from simulation to be 34 Ohms at ω_0 and 33 Ohms at $\omega_{\rm p}$.

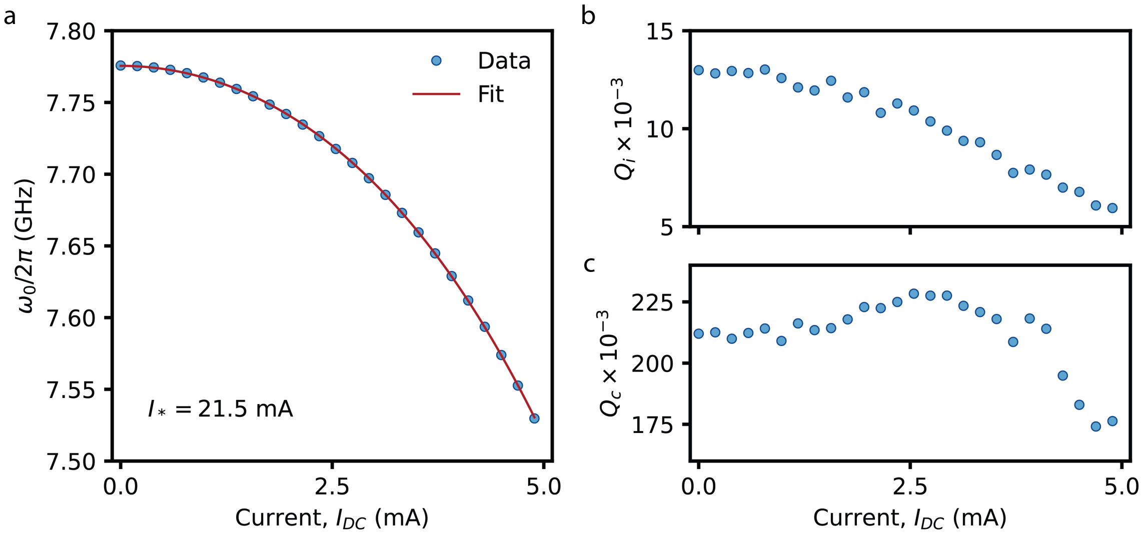

FIG. S5. The resonance frequency and quality factors measured as a function of $I_{\rm DC}$. The values are extracted for measurements of S_{11} using a VNA with a power such that $\bar{n} \approx 1$. The data in (a) is fit with Eq. S2 to extract the constant I_*.

III. MEASUREMENTS OF RESONATOR FREQUENCY AND Q-FACTOR

Measurements of S_{11} were performed with a Vector Network Analyzer (VNA) (Keysight PNA-L N5231B). Due to the narrow bandwidth of the resonator relative to its frequency tunability, measurements of S_{11} with the resonator far-detuned were subtracted to remove background ripple and correct for the line delay. For the lowest power measurements in Fig. 1D of the main text, the signal was digitally filtered with a Savitzky–Golay filter to improve the signal to noise ratio.

Fig. S5 shows ω_0, Q_i and Q_c measured as a function of $I_{\rm DC}$, which are extracted from fits of S_{11} to cavity input-output theory. The resonance frequency $\omega_0/2\pi$ could be tuned by 246 MHz by applying $I_{\rm DC} = 4.89$ mA (Fig. S5a). The kinetic inductance is expected to vary with I_{DC} according to

$$L_k(I_{\rm DC}) = L_k(0)\left[1 + \frac{I_{\rm DC}^2}{I_*^2} + \mathcal{O}(I^4)\right], \tag{S1}$$

where I_* is a constant [36]. This in turn results in the frequency of the resonator varying as [7, 36]

$$\omega_0(I_{\rm DC}) = \omega_0(0)\left[1 - \frac{I_{\rm DC}^2}{2I_*^2}\right]. \tag{S2}$$

Fitting the measured $\omega_0(I_{\rm DC})$ with Eq. S2 yields $I_* = 21.5$ mA for this device. Notably, the

device is extremely under-coupled over the entire range of operation, with Q_i/Q_c in the range $[0.033, 0.064]$ (Fig. S5b,c).

Fig. 1D of the main text shows measurements of Q_i taken as a function of the applied microwave power P_0. This can be converted to an average number of intracavity photons $\bar{n} = 2Q_L^2 P_0/(\hbar\omega_0^2 Q_c)$ where $Q_L = (Q_i^{-1} + Q_c^{-1})^{-1}$ is the loaded quality factor [37]. The general improvement of Q_i with $\bar{n}$ is consistent with many other studies of high-Q superconducting microwave resonators, where the influence of Two Level Systems (TLSs) on Q_i has been well documented [25]. Notably, where TLSs limit Q_i, one typically expects Q_i to flatten below $\bar{n} = 1$, because the microwave power is insufficient to depolarize the TLSs. This is not observed in the range of signal powers studied here. We note that some authors have reported that Q_i reduces with signal power down to $\bar{n} = 10^{-3}$ [38].

IV. MODEL OF A PARAMETRICALLY-PUMPED DUFFING OSCILLATOR

To understand the behaviour of the KIPA when strongly pumped, we seek to develop a theoretical model based on a parametrically-pumped Duffing oscillator, which has previously proven to be an excellent description for Josephson Parametric Oscillators [2, 23, 24]. For a classical oscillator with position x, the Duffing equation is given by

$$\ddot{x} + 2\lambda\dot{x} + \left(\omega_0^2 + F\cos(2\omega t)\right)x - dx^3 = 0, \tag{S3}$$

where λ is the linewidth of the oscillator, ω is the frequency of a periodic drive with strength F, and d is the Duffing constant. For a mass on a spring, the Duffing constant describes a softening or stiffening of the spring as it extends from its equilibrium position. We show below that for a superconducting microwave resonator it is related to the Kerr effect.

To relate the Duffing equation to the KIPA we take the approach of Lin, et al. [2] and begin with the master equation for the intracavity field, which is given by

$$\dot{a} = \frac{1}{i\hbar}[a, H_{\mathrm{KIPA}}] + \sqrt{\kappa}a_{\mathrm{in}}(t) + \sqrt{\gamma}b_{\mathrm{in}}(t) - \frac{\kappa+\gamma}{2}a. \tag{S4}$$

Here a ($a^\dagger$) is the bosonic annihilation (creation) operator for the cavity mode, a_{in} ($a_{\mathrm{in}}^\dagger$) is the bosonic annihilation (creation) operator for the port mode, and b_{in} ($b_{\mathrm{in}}^\dagger$) is the bosonic annihilation (creation) operator for the bath mode. $\kappa = \omega_0/Q_c$ and $\gamma = \omega_0/Q_i$ are the rates at which photons couple from the cavity mode to the port mode and bath mode, respectively.

The Hamiltonian for the KIPA was previously derived in Reference [7]. In the frame rotating at half the pump frequency it is given by

$$\frac{H_{\mathrm{KIPA}}}{\hbar} = \Delta a^\dagger a + \frac{\zeta}{2}a^{\dagger 2} + \frac{\zeta^*}{2}a^2 + \frac{K}{2}a^{\dagger^2}a^2, \tag{S5}$$

where

$$\Delta = (\omega_0 + \delta_{\mathrm{DC}} + \delta_{\mathrm{p}} + K - \frac{\omega_{\mathrm{p}}}{2}), \tag{S6}$$

$$\delta_{\mathrm{DC}} = -\frac{1}{2}\frac{I_{\mathrm{DC}}^2}{I_*^2}\omega_0, \tag{S7}$$

$$\delta_{\mathrm{p}} = -\frac{1}{8}\frac{I_{\mathrm{p}}^2}{I_*^2}\omega_0, \tag{S8}$$

$$K = -\frac{3}{8}\frac{\hbar\omega_0}{L_T I_*^2}\omega_0, \tag{S9}$$

$$\zeta = -\frac{1}{4}\frac{I_{\mathrm{DC}}I_{\mathrm{p}}}{I_*^2}\omega_0 e^{-i\phi_{\mathrm{p}}}. \tag{S10}$$

Here, Δ is a detuning of half of the pump frequency from the point of degeneracy (i.e. the cavity frequency), ζ is the three-wave mixing strength, and K is the Kerr strength. Δ accounts for the shift of the cavity with an applied DC current ($\delta_{\rm DC}$) and pump current ($\delta_{\rm p}$) due to the nonlinear kinetic inductance in the KIPA. I_* is a constant that sets the scale of the non-linearity of the KIPA and can be experimentally determined by measuring $\delta_{\rm DC}$ with a Vector Network Analyzer (VNA), as demonstrated in Fig. S5a. $I_{\rm DC}$ is the applied DC current that enables three-wave mixing and $I_{\rm p}$ is the pump current peak amplitude. L_T is the total kinetic inductance and $\phi_{\rm p}$ is the phase of the pump tone.

Substituting Eq. S5 into Eq. S4 yields

$$\dot{a} = -i\Delta a - i\zeta a^\dagger - iKa^\dagger a^2 - \frac{\kappa+\gamma}{2}a + \sqrt{\kappa}a_{\rm in}(t) + \sqrt{\gamma}b_{\rm in}(t). \tag{S11}$$

Next we consider the simplification $\langle a_{\rm in}\rangle = \langle b_{\rm in}\rangle = 0$, which corresponds to the case where no resonant signals are sent to the cavity via the port or bath modes. In this case, Eq. S11 can be transformed into two real-valued coupled differential equations by substituting $a = X - iY$, where X and Y are the operators for the quadrature amplitudes of the intracavity field. Doing so yields

$$\begin{pmatrix}\dot{X}\\ \dot{Y}\end{pmatrix} = \begin{pmatrix}-Y\Delta + Y\zeta - K(X^2+Y^2)B - \bar{\gamma}X,\\ X\Delta + X\zeta + K(X^2+Y^2)X - \bar{\gamma}Y\end{pmatrix}, \tag{S12}$$

where $\bar{\gamma} = (\kappa+\gamma)/2$. This can be recast in the form

$$\frac{1}{\bar{\gamma}}\frac{\partial}{\partial t}\begin{pmatrix}X\\ Y\end{pmatrix} = \begin{pmatrix}-X - \partial g(X,Y)/\partial Y\\ -Y + \partial g(X,Y)/\partial X\end{pmatrix}, \tag{S13}$$

where

$$g(X,Y) = \frac{\Delta}{2\bar{\gamma}}(X^2+Y^2) + \frac{\zeta}{2\bar{\gamma}}(X^2-Y^2) + \frac{K}{4\bar{\gamma}}(X^2+Y^2)^2. \tag{S14}$$

Previous authors have noted the similarity of Eq. S13 to Hamilton's equations [23, 24]. This has lead to Eq. S14 being described as a metapotential because the behaviour of the superconducting circuit is analogous to a particle traversing the potential surface $g(X,Y)$.

In Eq. S14, the terms $\Delta/(2\bar{\gamma})$, $\zeta/(2\bar{\gamma})$, and $K/(2\bar{\gamma})$ correspond generalized versions of the detuning of the pump frequency, the pump strength, and the Duffing non-linearity, respectively. Their influence on the behaviour of the oscillator become clear by taking the steady state solution of Eq. S13. We also make use of a change of variables for X and Y so that they can be described in terms of a single amplitude α and phase θ by making the substitutions $X = \alpha\cos(\theta)$ and $Y = \alpha\sin(\theta)$. Doing so yields

$$\cot(\theta) = \frac{1}{\bar{\gamma}}\left(\zeta + \Delta + \alpha^2 K\right), \tag{S15}$$

$$\tan(\theta) = \frac{1}{\bar{\gamma}}\left(\zeta - \Delta - \alpha^2 K\right). \tag{S16}$$

Equations S15 and S16 are π-periodic, and thereby imply the existence of two non-trivial solutions. Moreover, it is straightforward to solve for α by taking their product, which yields a solution that is independent of θ and is equal to

$$\alpha = \left(\pm\frac{\sqrt{\zeta^2-\bar{\gamma}^2} - \Delta}{K}\right)^{1/2}. \tag{S17}$$

Because α is an amplitude, we require Eq. S17 to yield real solutions. This is true only when

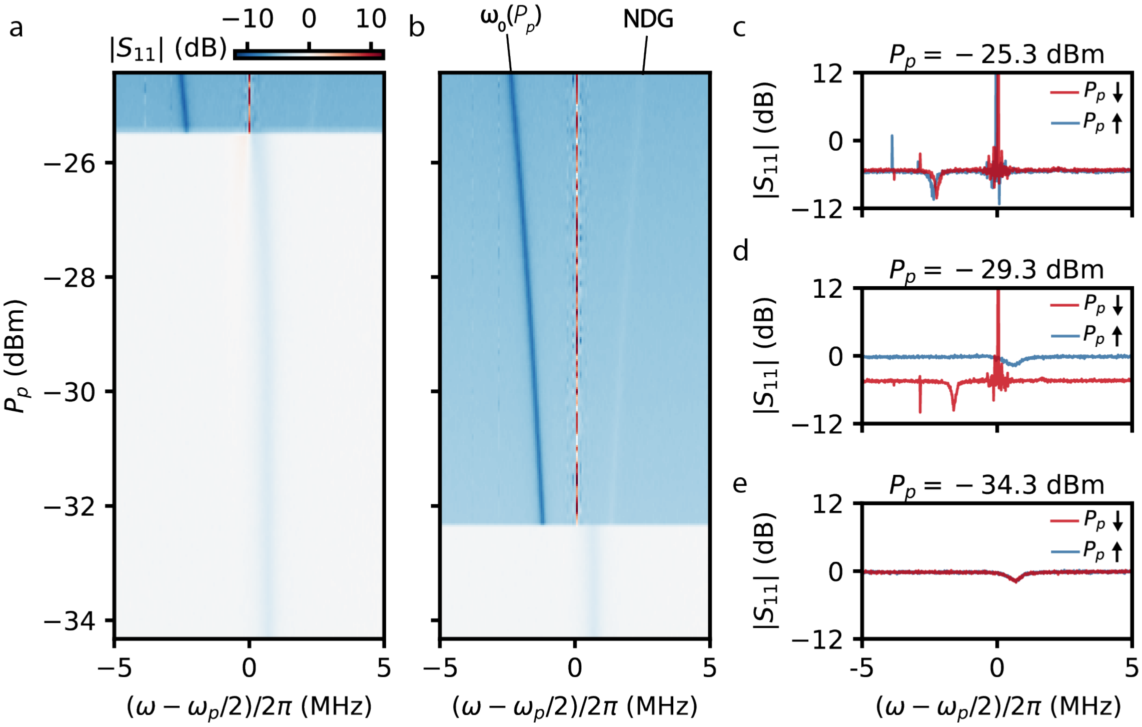

FIG. S6. Hysteresis of self-oscillations. (a) VNA measurements of $|S_{11}|$ as P_p is increased. (b) $|S_{11}|$ as P_p is decreased. The labels $\omega_0(P_\mathrm{p})$ and NDG show the shifted resonance frequency and the idler associated with a non-degenerate gain process, respectively. (c-e) Line cuts from (a) and (b) at various powers. The DC current was set to $I_\mathrm{DC} = 0.83$ mA for these measurements. The sharp change in the baseline power is due to the saturation of the amplification chain when the device self-oscillates. The colourmap for (a) was truncated at +12 dB and also applies to panel (b).

$$\zeta^2 > \Delta^2 + \bar{\gamma}^2, \tag{S18}$$

so that we can interpret Eq. S18 as a boundary in parameter space. When the inequality is false, the device functions as a linear parametric amplifier. In this case, provided $\langle a_\mathrm{in} \rangle = 0$, α will remain equal to zero. When the inequality is true, however, the resonator will quickly develop a large intracavity field with amplitude α and phase $\theta = 0$ or $\theta = 1\pi$.

V. HYSTERETIC PARAMETRIC SELF-OSCILLATIONS

In Fig. 2 of the main text it is demonstrated that the detector signal latches. This is related to the fact that Q_i is dependent upon $\bar{n}$, which results in hysteresis of P_th. This hysteresis can be directly observed when measuring $|S_{11}|$ with a VNA while sweeping P_p. In Fig. S6a we increase P_p and observe a sharp transition in the behaviour of the device as P_p is raised beyond -25.5 dBm. In contrast, when P_p is decreased, the behaviour changes at $P_\mathrm{p} = -32.4$ dBm (Fig. S6b).

Line cuts taken from the two measurements show that the device functions as a simple resonator for $P_\mathrm{p} < -32.3$ dBm (Fig. S6e) and as a parametric oscillator for $P_\mathrm{p} > -25.4$ dBm (Fig. S6c). The behaviour observed at large P_p can be clearly identified as parametric self-oscillations due to the large power generated at exactly half the pump frequency. For P_p intermediate to these values, the device functions as a parametric amplifier prior to latching. Notably, the linear parametric gain achieved is much smaller than in previous studies using KIPAs [7, 10], which is a consequence of the fact that $Q_i < Q_c$ for this device. The power generated in the self-oscillating state is large enough to cause the amplification chain to compress, which results in the baseline shifting by -5.6 dB. For a parametric oscillator, one would also expect that the amplitude of the self-oscillations would be arrested by a non-linear shift in the resonance frequency due to the Duffing

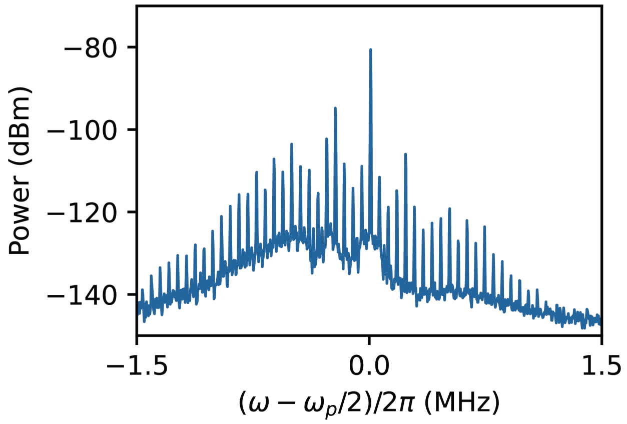

FIG. S7. A spectrum of the self-oscillation signal. For this measurement $I_{\rm DC} = 2.5$ mA and the resonator frequency was measured to be $\omega_0/2\pi = 7.7203$ GHz. The only signals supplied to the device during the measurement are the DC current $I_{\rm DC}$ and a pump with frequency $\omega_p = 2\omega_0$ and power $P_{\rm p} = -50.3$ dBm. All powers are referred to the device input.

non-linearity. This is because for a fixed $\omega_{\rm p}$ and $P_{\rm p}$, shifting ω_0 will reduce the rate of down-conversion, which in combination with a finite cavity bandwidth set by Q_L, eventually leads the intracavity field to a high-amplitude equilibrium. Fig. S6b clearly reveals this behaviour, with the detuning $(\omega_{\rm p}/2 - \omega_0(P_{\rm p}))/2\pi$ reaching a maximum of 3.2 MHz at the highest $P_{\rm p}$ measured.

It is also notable that the depth of the resonance appears to increase when the device self-oscillates. Fitting the resonance in isolation from the other features confirms this and reveals that it is primarily the result of Q_i, which increases from 13×10^3 to 50×10^3 when the device self-oscillates. We attribute this to be the result of the down-converted power partially saturating the TLSs.

VI. EVIDENCE OF ADDITIONAL MIXING PROCESSES

The measurements of S_{11} shown in Fig. S6 reveal several features that are not expected from the basic theory of a parametric oscillator, namely several sharp peaks on the red sideband and fine structure near $\omega = \omega_{\rm p}/2$. To better understand these features, we measure the output signal with a spectrum analyser centered on ω_0 (as measured with a VNA with the pump off) when the device is supplied only a DC current and a pump with frequency $\omega_p = 2\omega_0$. Fig. S7 shows one spectrum obtained with $I_{\rm DC} = 2.5$ mA and $P_{\rm p} = -50.3$ dBm. For this setpoint, $P_{\rm p}$ is just above the level required to initiate parametric self-oscillations, but similar results are obtained for larger $P_{\rm p}$ and for different $I_{\rm DC}$. The spectrum reveals a frequency comb that spans nearly the entire 3 MHz bandwidth of the measurement, with teeth that are equally spaced by 54 kHz. The tooth of the comb with the largest power is indeed centered at $\omega_p/2$, as expected for a parametric oscillator. The generation of frequency combs with superconducting microwave resonators has been demonstrated in several works including References [39–43] with several mechanisms underlying comb formation. We also note that the spectrum is obtained with phase modulation of the pump disabled, and direct measurements of the microwave sources do not reveal any sidebands that might otherwise explain the frequency comb generated by the KIPO. While a full explanation of the comb generated by the KIPO is beyond the scope of this work, it nevertheless has important implications to the experiments of this manuscript. First, when the KIPO is made to self-oscillate, the combs introduce a time-varying component to the amplitude of the signal demodulated with a local oscillator of frequency $\omega_{\rm LO} = \omega_p/2$, as in Figs. 2b and 4b of the main text. Second, the equal spacing of the teeth suggests they are likely generated via higher-order mixing processes (e.g. four wave mixing), which would thereby deplete power from the tooth at $\omega_p/2$. As the frequency comb is not captured in the model of a Duffing oscillator used to describe the KIPO, the amplitude of the self-oscillating state given by the model (Eq. S17) will not match that of the experiments. Because the frequency comb is generated only upon the initiation of parametric self-oscillations, however, the Duffing oscillator model nevertheless remains applicable for estimating the parametric

self-oscillation threshold $P_{\rm th}(P_{\rm p}, \omega_p)$.

VII. MAPPING THE SELF-OSCILLATION BOUNDARY

In this section we show how $P_{\rm th}$, which is written in terms of the KIPA Hamiltonian in Eq. S18, can be compared to experiments.

We begin by expanding the Δ^2 term using Eq. S6, which yields

$$\begin{aligned}\Delta^2 &= \omega_0^2 + \delta_{\rm DC}^2 + \delta_{\rm p}^2 + K^2 + \frac{\omega_{\rm p}^2}{4} \\ &+ 2\omega_0\left(\delta_{\rm DC} + \delta_{\rm p} + K - \frac{\omega_{\rm p}}{2}\right) \\ &+ 2\delta_{\rm DC}\left(\delta_{\rm p} + K - \frac{\omega_{\rm p}}{2}\right) \\ &+ 2\delta_{\rm p}\left(K - \frac{\omega_{\rm p}}{2}\right) - K\omega_{\rm p}.\end{aligned} \tag{S19}$$

To simplify this we make note of the relative scales of each term. $\omega_0/2\pi$ and $\omega_{\rm p}/2\pi$ are both of order GHz. For our experiments, $\delta_{\rm DC}/2\pi$ is tens of MHz. The expression for $\delta_{\rm p}$ is given by Eq. S8 where it is written in terms of $I_{\rm p}^2$. To get a sense of its magnitude we can write it in terms of a microwave power $P_{\rm p}$ by substituting $I_{\rm p}^2 \to 2P_{\rm p}/Z_{\rm p}$, where $Z_{\rm p}$ is the impedance of the device at frequency $\omega_{\rm p}$. From simulations of the device (Sonnet) we expect $Z_{\rm p} \approx 33$ Ohms. For $P_{\rm p} = -33$ dBm, the largest power used for the experiment shown in Fig. 1C of the main text, this yields $\delta_{\rm p}/2\pi \approx 64$ kHz. From previous works we know that $K/2\pi$ is of order Hz for these devices [7]. We therefore conclude that $\omega_0, \omega_{\rm p} \gg \delta_{\rm DC} \gg \delta_{\rm p} \gg K$ which allows us to approximate Eq. S19 as

$$\Delta^2 \approx \underbrace{\omega_0^2 + \frac{\omega_{\rm p}^2}{4} - \omega_0\omega_{\rm p}}_{\rm GHz^2} + \underbrace{2\omega_0\delta_{\rm DC} - \omega_{\rm p}\delta_{\rm DC}}_{\rm GHz\times MHz} + \underbrace{2\omega_0\delta_{\rm p} - \omega_{\rm p}\delta_{\rm p}}_{\rm GHz\times kHz} + \underbrace{\delta_{\rm DC}^2}_{\rm MHz^2}, \tag{S20}$$

where we have omitted the terms which are lower in frequency.

Here we note that in the main text we define $\Delta_{\rm p} = \omega_{\rm p} - 2\omega_0(I_{\rm DC})$, where $\omega_0(I_{\rm DC})$ is an experimentally measured value of the resonant frequency with an $I_{\rm DC}$ applied. Written in terms of Eqs. S7-S9 this is

$$\Delta_{\rm p} = \omega_{\rm p} - 2\underbrace{(\omega_0 + \delta_{\rm DC})}_{\omega_0(I_{\rm DC})} - \underbrace{2(\delta_{\rm p} + K)}_{\approx 0} \approx \omega_{\rm p} - 2(\omega_0 + \delta_{\rm DC}), \tag{S21}$$

where we neglect the small shift in the resonance frequency due to the microwave power of the tone used to measure S_{11}. This is an important difference given that Δ accounts for the shift $\delta_{\rm p}$ due to the pump power, whereas $\Delta_{\rm p}$ does not. Using the approximation in Eq. S20 we find

$$\Delta^2 \approx \left(\frac{\Delta_{\rm p}}{2}\right)^2 + \delta_{\rm p}(2\omega_0 - \omega_{\rm p}) \tag{S22}$$

$$= \left(\frac{\Delta_{\rm p}}{2}\right)^2 - \delta_{\rm p}(\Delta_{\rm p} + 2\delta_{\rm DC}). \tag{S23}$$

Here we see that Eq. S23 contains a term that is proportional to $\delta_{\rm p}$ that accounts for the shift of the resonant frequency with the pump power.

Using Eqs. S6-S10 and Eq. S23, Eq. S18 can be written as

$$\zeta^2 > \left(\frac{\Delta_\text{p}}{2}\right)^2 - \delta_\text{p}(\Delta_\text{p} + 2\delta_\text{DC}) + \bar{\gamma}^2 \tag{S24}$$

$$\frac{1}{16}\frac{I_\text{DC}^2 I_\text{p}^2}{I_*^4}\omega_0^2 e^{-2i\phi_\text{p}} > \left(\frac{\Delta_\text{p}}{2}\right)^2 + \frac{1}{8}\frac{I_\text{p}^2}{I_*^2}\omega_0\left(\Delta_\text{p} - \frac{I_\text{DC}^2}{I_*^2}\omega_0\right) + \frac{\omega_0^2}{4}(Q_i^{-1} + Q_c^{-1})^2. \tag{S25}$$

We then solve for I_p^2 and convert it to a pump power as above. Without loss of generality, we assume ζ to be real and a positive value, i.e. we ignore the phase ϕ_p, which is only relevant when a coherent signal is being amplified, whereas here we consider only vacuum noise. We also substitute $Z_\text{p} \to \alpha_\text{p} Z_\text{p}$, where α_p is a constant that accounts for ripple in the transmission of the pump power through the device. This yields

$$P_\text{p} > P_\text{th} = \frac{\alpha_\text{p} Z_\text{p}}{2}\frac{\left(\frac{\Delta_\text{p}}{2}\right)^2 + \frac{\omega_0^2}{4}(Q_i^{-1} + Q_c^{-1})^2}{\frac{3}{16}\frac{I_\text{DC}^2\omega_0^2}{I_*^4} - \frac{1}{8}\frac{\omega_0\Delta_\text{p}}{I_*^2}}. \tag{S26}$$

For the models shown in Fig. 1C of the main text we set $Q_c = 220 \times 10^3$ and $I_* = 21.5$ mA based on the measurements shown in Fig. S5. We set $Z_\text{p} = 33$ Ohms based on simulations of the device (Sonnet) and $\alpha_\text{p} = 1.3$ (1.13 dB) because it reproduces the experimental data well (the width of the self-oscillating region is primarily set by $\alpha_\text{p} Z_\text{p}$). With other KIPAs we have observed that α_p can vary by as much as 10 dB over the operating frequency range (the ripple can can be inferred from measurements of the P_p required to achieve a fixed gain while tuning $\omega_0(I_\text{DC})$). We also note that in Fig. 1C we apply an offset of $\Delta_\text{p}/2\pi = -500$ kHz to the x-axis to center the parameter region where we observe parametric self-oscillations on $\Delta_\text{p} = 0$; this likely indicates that the frequency of the device drifted between the times Fig. 1C and $\omega_0(I_\text{DC})$ were measured.

Overall, the model qualitatively reproduces the self-oscillation boundary that is measured. The main effect of increasing Q_i is to shift the boundary to lower P_p. Good agreement between the model and the data occurs when $Q_i = 18 \times 10^3$. This is notable because $Q_i(\bar{n} = 0.01) \approx 5 \times 10^3$ is measured when the pump is off (Fig. 1D of the main text). This might indicate that our model is incomplete, or that Q_i is not independent of P_p, as we have assumed thus far. Supporting the latter hypothesis is the recent work by Qiu, et al. who found that it was essential to consider the P_p-dependent saturation of the TLSs due to amplified vacuum noise to quantitatively explain the behaviour of their amplifier [44].

The main takeaway we highlight is that the model gives reasonable estimates for the observed P_th. Moreover, because the device is undercoupled, its linewidth, and hence P_th, is very sensitive to $\bar{n}$. This is the basis of our detector: P_th can be dynamically reduced below a fixed P_p when the device absorbs resonant power, resulting in onset of parametric self-oscillations.

VIII. PHASE-COHERENT MEASUREMENTS RESOLVING THE QUIET, 0π, AND 1π STATES

So far, our measurements have shown that when $P_\text{p} > P_\text{th}$ the device generates a high-amplitude field at frequency $\omega_\text{p}/2$. Central to the model of a parametrically driven Duffing oscillator, however, is that there are two phase-coherent self-oscillating states, referred to as the 0π and 1π states.

To directly resolve these states, we down-convert the signal emitted from the device using a mixer driven by a local oscillator with frequency $\omega_\text{LO} = \omega_\text{p}/2$ and digitize the resulting quadrature amplitudes, $X(t)$ and $Y(t)$. In Fig. S8b we show a histogram of the signal in the XY-plane when the device is pumped with $P_\text{p} < P_\text{th}$. This is the quiet state of a parametric oscillator and corresponds to amplified vacuum noise; it is therefore a circle that rests at the origin of the XY-plane. In Fig. S8c we set $P_\text{p} > P_\text{th}$ and observe the emergence of two high-amplitude phase-coherent states. These are the 0π and 1π states. Their phase with respect to one another is fixed and equal to π, but their specific orientation in the XY-plane is set by their phase with respect to the local oscillator. We therefore choose to align both states along the Y axis during post-processing. We note that to observe both the 0π and 1π states requires measuring the system many times. The histograms in Fig. S8b,c correspond to 250 ms of digitized data, which was acquired from 500 shots of the

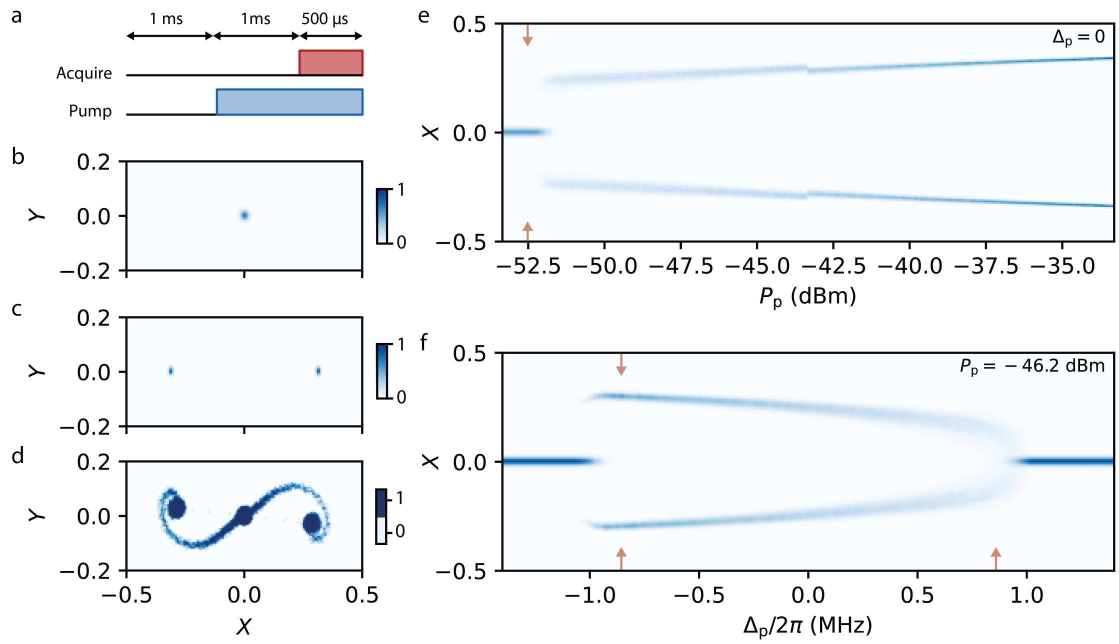

FIG. S8. Histograms of demodulated self-oscillations. (a) The pulse sequence used to measure the histograms. It is repeated 500 times for each measurement. (b) A histogram where $P_{\mathrm{p}} < P_{\mathrm{th}}$, showing only the quiet state. (c) A histogram where $P_{\mathrm{p}} > P_{\mathrm{th}}$, showing the 0π and 1π states. (d) A histogram where $P_{\mathrm{p}} \approx P_{\mathrm{th}}$, showing the transition from the quiet to the self-oscillating states. Note that for this panel the histogram values are binary so the trajectories between the states can be easily seen. (e) A series of histograms in the XY-plane that have been projected onto X, showing the population evolve from the quiet state to the 0π and 1π states as P_{p} is increased. The red arrows correspond to P_{th} predicted from our model. (f) The same as in (e), except Δ_{p} is varied for a fixed $P_{\mathrm{p}} = -46.2$ dBm. The red arrows correspond to the Δ_{p} at which the model predicts the onset of parametric self-oscillations.

pulse sequence depicted in Fig. S8a. The pulse sequence provides 1 ms of dead time to ensure the device resets, followed by 1 ms where the pump is turned on, and then 500 μs where the pump remains on and the demodulated signal is digitized. The 1 ms period where the pump is turned on prior to digitizing the signal is intended to allow the device to reach its steady state. Nevertheless, when $P_{\mathrm{p}} \approx P_{\mathrm{th}}$, occasionally the device is seen to transition from the quiet state to either the 0π or 1π states. These transitions are effectively irreversible while the pump remains on, due to the hysteresis observed in Fig. S6. These transitions can be seen clearly by renormalizing the colormap so that every bin is either zero (no counts) or one (one or more counts), which show that the trajectories taken from the quiet to the 0π and 1π states are coherent and reproducible (Fig. S8d).

It is important to ensure no resonant power is supplied to the device during these measurements. It is well known that even a weak tone can cause the device to favour the 0π or 1π states, a phenomenon commonly referred to as injection locking [2]. In the present experiments, the balanced weighting of the 0π and 1π states confirms injection locking is not occurring.

By acquiring a series of these histograms, we can map the time-averaged populations of the quiet, 0π, and 1π states as a function of experimental parameters. In Figs. S8e,f we show series of histograms measured as a function of P_{p} and Δ_{p}, respectively. These measurements are equivalent to taking vertical and horizontal line-cuts across the self-oscillation boundary, which is mapped in Fig. 1C of the main text. For both sets of measurements, the self-oscillation boundary as predicted by the model described above is indicated by red arrows and shows good agreement with the measurements. The device parameters used in the model are the same as in Fig. 1C, with $Q_i = 18 \times 10^3$. Note that as we did for Fig. 1C, we apply here an offset of $\Delta_p = -400$ kHz to account for drift in the resonance frequency.

While the amplitude of the self-oscillating states increases with P_{p} and decreases with Δ_{p}, as broadly predicted by the model in Eq. S17, we note that the model does not produce a quantitative match to this aspect of our experiments. As stated above, this is because the model does not

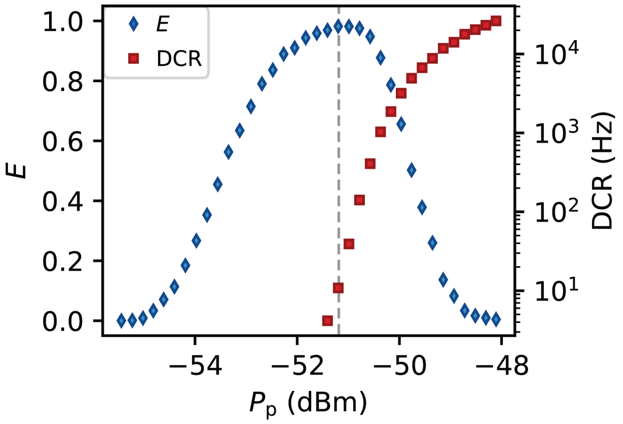

FIG. S9. KIPO dark count rate. A comparison between the detection efficiency E and DCR. The data is from the same experiment as shown in Fig. 3C of the main text, where E was measured for pulses with duration $\tau_1 = 10$ μs and power $P_0 = -111$ dBm. The vertical dashed line corresponds to $P_\mathrm{p} = -51.2$ dBm, which is the P_p used in Fig. 3E-G of the main text.

account for the emergence of the frequency comb shown in Fig. S7 which deplete the power at $\omega_\mathrm{p}/2$. Future refinements to the model, such as the inclusion of additional mixing processes and a P_p-dependent Q_i, may facilitate a quantitative match to all aspects of our experiments.

IX. DETECTOR DARK COUNT RATE

In Fig. 3D of the main text we show the probability of measuring dark counts $P(T|\tilde{S})$ as a function of the pump power P_p. From this measurement we also determine the dark count rate (DCR). To do so, we make use of the fact that the detector latches in the self-oscillating state. We can then calculate the DCR as

$$\mathrm{DCR} = \frac{n}{N\tau_\mathrm{tot} - \sum_i^n(\tau_\mathrm{tot} - t_i)} \tag{S27}$$

where $\tau_\mathrm{tot} = \tau_0 + \tau_1 + \tau_2 = 120$ μs is the total duration of the pump pulse in each shot of the control experiment (Fig. 3B of the main text), $N = 10^4$ is the total number of shots of the control pulse sequence (depicted in Fig. 3B of the main text), n is the number of dark counts, and t_i is the time at which the detector "clicks" measured from the rising edge of the pump pulse.

In Fig. S9 we plot the DCR as a function of P_p. We also directly compare it to the detector efficiency E measured during the same experiment with pulses of duration $\tau_1 = 10$ μs and power $P_0 = -111$ dBm. The minimum dark count rate we can measure is limited by the total measurement time and is equal to $1/(N\tau_\mathrm{tot}) = 0.83$ Hz. For $P_\mathrm{p} < -51.4$ dBm we detect no dark counts, whereas for $P_\mathrm{p} > -50.4$ dBm the DCR exceeds 1 kHz.

In Figs. 3e-g of the main text we determine the detection sensitivity and demonstrate that E is phase-sensitive. For these experiments we set $P_\mathrm{p} = -51.2$ dBm, where in Fig. S9 we measure a total of $n = 13$ dark counts in $N = 10^4$ shots, corresponding to a DCR of 10.8 Hz.

X. RECEIVER OPERATING CHARACTERISTIC CURVE

There are a wide variety of metrics that can be used to benchmark the performance of a detector. Throughout the main text we use the detector efficiency $E = P(T|S) - P(T|\tilde{S})$ as a single metric. One disadvantage of this approach, however, is that by itself E does not communicate the specific balance between the probability of successful detection $P(T|S)$ and the probability of dark counts $P(T|\tilde{S})$, both of which may vary with different settings of the detector. A common approach to evaluating these trade-offs is to plot an receiver operating characteristic (ROC) curve, which

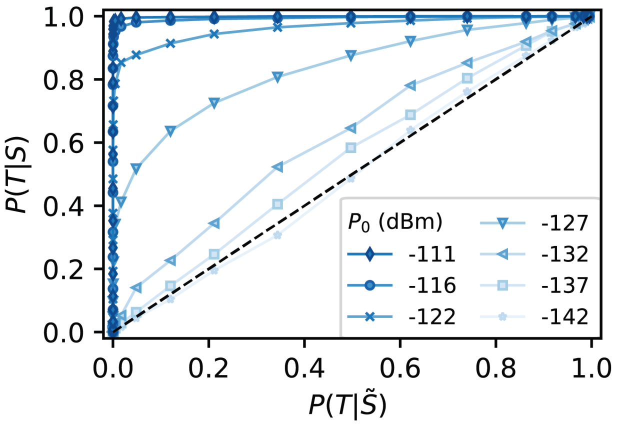

FIG. S10. Receiver operating characteristic curve. The data is the same as is shown in Fig. 3C of the main text. A black diagonal line is plotted along the diagonal $P(T|S) = P(T|\tilde{S})$ and corresponds to a random binary classifier.

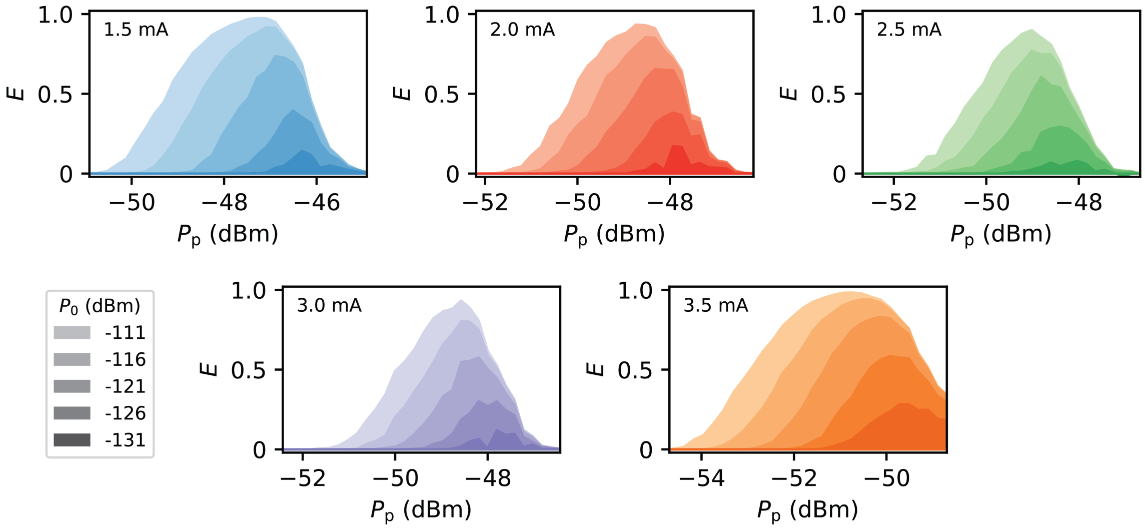

FIG. S11. The E measured as a function of P_p for a range of I_{DC} setpoints. Each datapoint corresponds to 5×10^3 shots of both pulse sequences depicted in Figs. 3a,b of the main text, with $\tau_1 = 10$ μs. The legend in the lower left corner applies to all panels.

compares $P(T|S)$ directly to $P(T|\tilde{S})$. In Fig. S10 we show an ROC curve for the experiment depicted in Fig. 3C of the main text. The black diagonal line corresponds to the scenario where $P(T|S) = P(T|\tilde{S})$, in which case the detector behaves as a random binary classifier, i.e. using the detector is equivalent to guessing. For the pulses with power $P_0 = -137$ dBm, the detector outperforms the random binary classifier. For this experiment, the pulses had duration $\tau_1 = 10$ μs, so that the total energy within the wavepacket was $J = P_0\tau_1 = 0.21^{0.27}_{0.17}$ zJ (42^{52}_{33} photons), where the upper and lower values correspond to a 1 dB uncertainty in P_0.

XI. DETECTOR EFFICIENCY WITH BIAS CURRENT

In Fig. S11 we compare measurements of E completed over a series of I_{DC} setpoints, corresponding to a tunable frequency range of $\delta_{DC}/2\pi \approx 95$ MHz. For each setpoint, the device achieves E near unity for pulses with duration $\tau_1 = 10$ μs and $P_0 = -111$ dBm. The similar performance of the device at each setpoint indicates it is generally insensitive to I_{DC}, and can therefore be used as a detector over much of its total tunable frequency range.

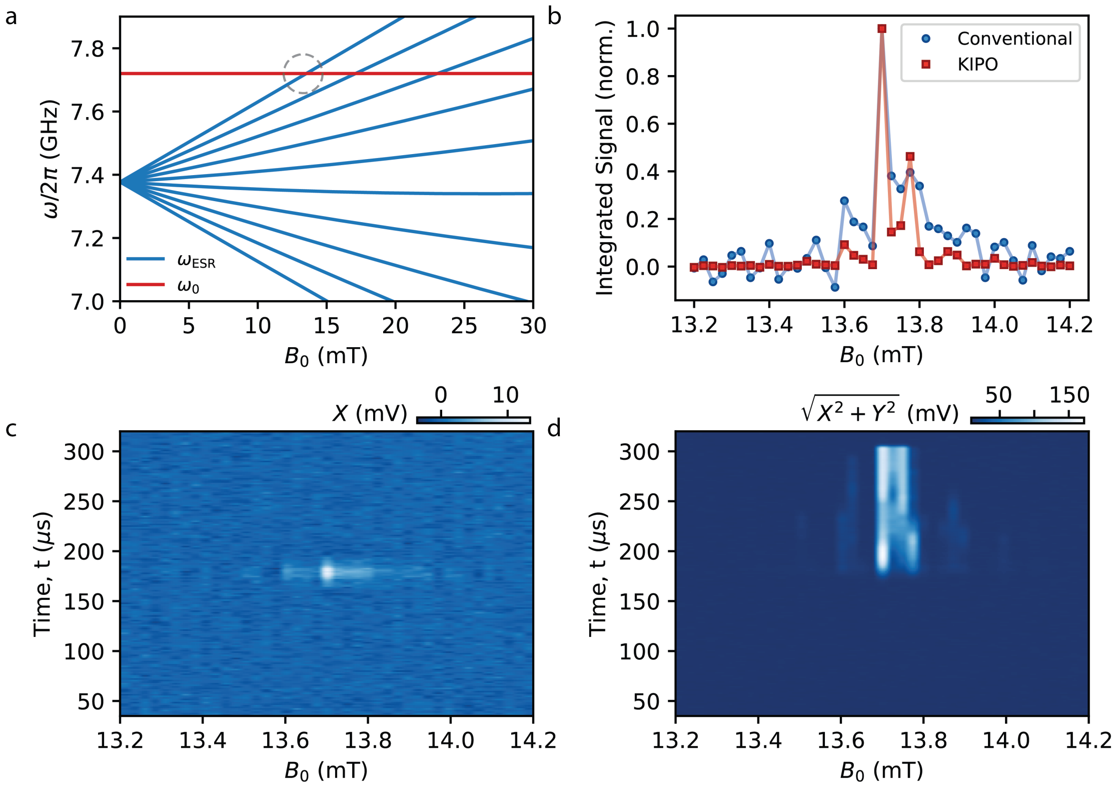

FIG. S12. Spectroscopy with CPMG measurements. (a) The frequency of the allowed ^{209}Bi ESR transitions (blue lines) as a function of magnetic field strength B_0. The dashed grey circle corresponds to the point where we perform ESR spectroscopy by matching the $|4,4\rangle \leftrightarrow |5,5\rangle$ spin transition frequency to the KIPO frequency (red line). (b) The integrated spin echo signal for a conventional CPMG measurement (blue circles) and using the KIPO (red squares). The two datasets are independently normalized. (c) The homodyne-demodulated signal for the conventional CPMG measurement sequence. The data in (b) is generated by integrating the spin echo signal, which appears as a high amplitude (bright) wavepacket, between $160 < t < 190$ μs. (d) The same as (c), but measured using the KIPO, with $P_{\mathrm{p}} = -49.2$ dBm. In both measurements we perform twenty shots of the CPMG-N sequence with $N = 20$ refocusing pulses and average the entire record. Averaging is justified, even for the KIPO measurements, because no dark counts were recorded for the control sequence (where the sequence omitting the $x_{\pi/2}$ pulse was used). The measurements in both panels were taken during the same field sweep, with $I_{\mathrm{DC}} = 2.53$ mA and a waiting period of 7 s between each shot of each pulse sequence.

XII. MEASURING ESR AS A FUNCTION OF MAGNETIC FIELD

To simulate the use of the KIPO as a spectroscopic probe, we measure ESR as a function of the magnetic field strength B_0. In Fig. S12a we show the allowed ^{209}Bi spin transition frequencies as a function of B_0, which are calculated numerically using the spin Hamiltonian $H = H_A + H_B$ described in the main text. We perform ESR at the point indicated by a dashed grey circle, where the $|4,4\rangle \leftrightarrow |5,5\rangle$ transition frequency matches the resonant frequency of the KIPO ω_0. In Fig. S12b, we compare the integrated spin echo signals measured with a conventional CPMG measurement (blue circles) and the modified CPMG measurement sequence where the spin echoes trigger parametric self-oscillations (red squares). Both techniques produce similar spectra, with a primary peak at 13.70 mT, a secondary peak at 13.78 mT, and two smaller satellites. We note that similar split-peaks have been observed in cryogenic micro-resonator measurements of ^{209}Bi donors before, where they were identified to be the result of strain [34]. The signal-to-noise ratio (SNR) for the data measured using the KIPO appears much greater in comparison to the conventional measurement, where noise is dominated by the HEMT amplifier at 4 K. This can be seen directly in the averaged data from which we derive the integrated signals, which are shown in Figs. S12c,d. These datasets correspond to the amplitude of the homodyne-demodulated signals.

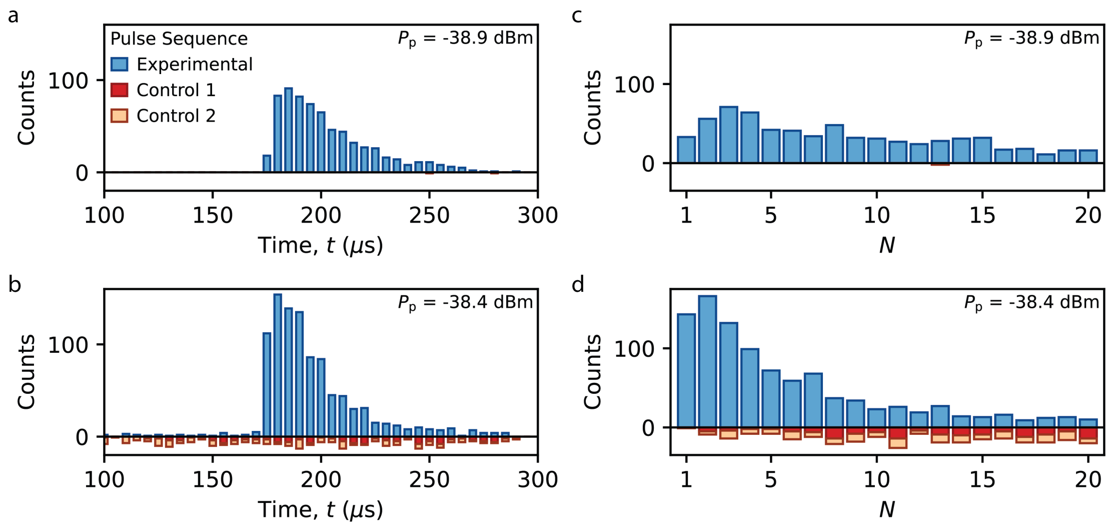

FIG. S13. Histograms of detector "clicks" for a CPMG measurement. (a,b) The distribution of "clicks" with time t for two P_p. The counts associated with the two control experiments are summed and plotted as negative values, so that they can be easily compared with the counts from the experimental sequence. (c,d) The distribution of clicks with refocusing pulse repetition N. The data is from the experiment shown in Fig. 4C of the main text.

For the conventional CPMG measurement, we show the signal measured on X, the quadrature onto which the spin echo signal was emitted. The self-oscillation signal that is triggered by the spin echoes cannot be aligned onto a single quadrature, so we instead plot the amplitude of the full homodyne-demodulated signal $\sqrt{X^2+Y^2}$.

XIII. HISTOGRAMS OF DETECTOR "CLICKS" IN A CPMG ESR MEASUREMENT

In Figs. S13a,b we plot histograms showing the time within the CPMG pulse sequence at which the detector "clicks." The data is from the same experiment shown in Fig. 4C of the main text where $N = 20$ refocusing pulses were used, and corresponds to the minimum and maximum values of P_p used in that experiment. For each shot of the pulse sequence, we consider only the first time the detector "clicks," because the amplitude of the self-oscillating state is sufficiently large to drive the spin system and scramble the echoes. For both P_p, the "clicks" of the experimental pulse sequence (blue bars) are triggered within a short time window near $t = 170$ μs, corresponding to the time at which the spin echo refocuses (see the inset of Fig. 4B of the main text). In contrast, the dark counts for the two control experiments (red and orange bars, plotted as negative values) show no trend with t. These histograms give further confidence that the "clicks" triggered in the experimental pulse sequence are indeed caused by spin echoes and are not an artifact of the measurement sequence.

In Figs. S13c,d we compare the distribution of the "clicks" with the repetition N, i.e. on which of the refocusing y_π pulses the detection is made. The data clearly reveals that for larger P_p, the "clicks" occur more frequently at smaller N. This is intuitive, as it suggests that as P_p approaches P_th, the sensor is more likely to "click" on any given repetition, in accordance with the model described in Section XIV.

XIV. SCALING OF SPIN DETECTION EFFICIENCY WITH N

In Figs. 4c,d of the main text we demonstrate that the detection efficiency E of a spin echo signal obtained with a CPMG pulse sequence employing N refocusing pulses can be modeled by the equation

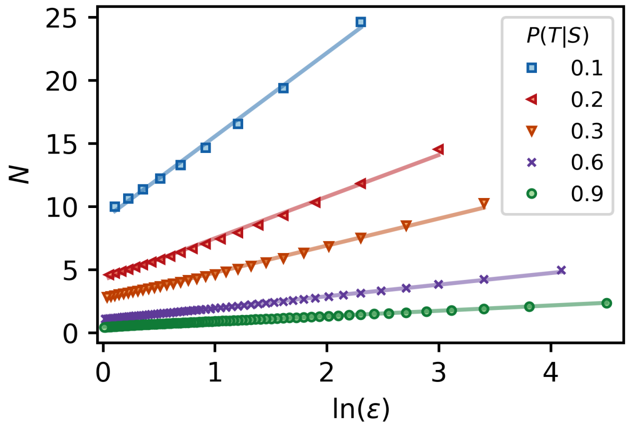

FIG. S14. The number of refocusing pulses N that maximizes the detection efficiency E in a CPMG experiment. The points are calculated using Eq. S30. The solid lines are linear fits of N as a function of $\ln(\epsilon)$.

$$E(N) = [1 - P(T|\tilde{S})]^N - [1 - P(T|S)]^N. \tag{S28}$$

Here $P(T|S)$ and $P(T|\tilde{S})$ are the probabilities associated with measuring a "click" with the experimental and control pulse sequences in a Hahn ($N = 1$) echo experiment, respectively. From this equation we can calculate the N at which E is maximized by setting $\partial E(N)/\partial N = 0$ and solving for N. This yields

$$\frac{\partial E(N)}{\partial N} = [1 - P(T|\tilde{S})]^N \ln[1 - P(T|\tilde{S})] - [1 - P(T|S)]^N \ln[1 - P(T|S)] = 0, \tag{S29}$$

$$N = -\frac{\ln\left(\frac{\ln[1-P(T|\tilde{S})]}{\ln[1-P(T|S)]}\right)}{\ln[1 - P(T|\tilde{S})] - \ln[1 - P(T|S)]}. \tag{S30}$$

In Fig. S14 we plot Eq. S30 as a function of the ratio $\epsilon = P(T|S)/P(T|\tilde{S})$ for several fixed values of $P(T|S)$. The solid lines are linear fits of N as function of $\ln(\epsilon)$, and capture the points well. This shows that the number of refocusing pulses N in a CPMG experiment required to maximize the detection efficiency E scales as $\mathcal{O}[\ln(\epsilon)]$.

REFERENCES AND NOTES

1. I. Siddiqi, R. Vijay, F. Pierre, C. M. Wilson, M. Metcalfe, C. Rigetti, L. Frunzio, M. H. Devoret, RFdriven Josephson bifurcation amplifier for quantum measurement. *Phys. Rev. Lett.* **93**, 207002 (2004).

2. Z. R. Lin, K. Inomata, K. Koshino, W. D. Oliver, Y. Nakamura, J. S. Tsai, T. Yamamoto, Josephson parametric phase-locked oscillator and its application to dispersive readout of superconducting qubits. *Nat. Commun.* **5**, 4480 (2014).

3. P. Krantz, A. Bengtsson, M. Simoen, S. Gustavsson, V. Shumeiko, W D Oliver, C M Wilson, P. Delsing, J. Bylander, Single-shot read-out of a superconducting qubit using a Josephson parametric oscillator. *Nat. Commun.* **7**:11417 (2016).

4. K. Petrovnin, J. Wang, M. Perelshtein, P. Hakonen, G. S. Paraoanu, Microwave photon detection at parametric criticality. *arXiv*. 2308.07084. (2023).

5. B. H. Eom, P. K. Day, H. G. Le Duc, J. Zmuidzinas, A wideband, low-noise superconducting amplifier with high dynamic range. *Nat. Phys.* **8**, 623–627 (2012).

6. N. Samkharadze, A. Bruno, P. Scarlino, G. Zheng, D. P. Di Vincenzo, L. Di Carlo, L. M. K. Vandersypen, High-kinetic-inductance superconducting nanowire resonators for circuit QED in a magnetic field. *Phys. Rev. Applied*, **5**, 044004 (2016).

7. J. G. Kroll, F. Borsoi, K. L. van der Enden, W. Uilhoorn, D. de Jong, M. Quintero-Pérez, D. J. van Woerkom, A. Bruno, S. R. Plissard, D. Car, E. P. A. M. Bakkers, M. C. Cassidy, L. P. Kouwenhoven Magnetic-Field-Resilient superconducting coplanar-waveguide resonators for hybrid circuit quantum electrodynamics experiments. *Phys. Rev. Applied* **11**, 064053, (2019).

8. A. Vaartjes, A. Kringhøj, W. Vine, T. Day, A. Morello, J. J. Pla, Strong microwave squeezing above 1 tesla and 1 kelvin. *arXiv*. 2311.07968. (2023).

9. D. J. Parker, M. Savytskyi, W. Vine, A. Laucht, T. Duty, A. Morello, A. L. Grimsmo, J. J. Pla, Degenerate parametric amplification via three-wave mixing using kinetic inductance. *Phys. Rev. Applied* **17**, 034064 (2022).

10. M. Xu, R. Cheng, Y. Wu, G. Liu, H. X. Tang, Magnetic field-resilient quantum-limited parametric amplifier. *PRX Quantum* **4**, 010322 (2023).

11. M. Khalifa, J. Salfi, Nonlinearity and parametric amplification of superconducting nanowire resonators in magnetic field. *Phys. Rev. Applied* **19**, 034024 (2023).

12. W. Vine, M. Savytskyi, A. Vaartjes, A. Kringhøj, D. Parker, J. Slack-Smith, T. Schenkel, K. Mølmer, J. C. Mc Callum, B. C. Johnson, A. Morello, J. J. Pla *et al.*, In-situ amplification of spin echoes within a kinetic inductance parametric amplifier. *Sci Adv* **9**, adg1593 (2023).

13. K. M. Backes, D. A. Palken, S. A. Kenany, B. M. Brubaker, S. B. Cahn, A. Droster, G. C. Hilton, S. Ghosh, H. Jackson, S. K. Lamoreaux, A. F. Leder, K. W. Lehnert, S. M. Lewis, M. Malnou, R. H. Maruyama, N. M. Rapidis, M. Simanovskaia, S. Singh, D. H. Speller, I. Urdinaran, L. R. Vale, E. C. van Assendelft, K. van Bibber, H. Wang, A quantum-enhanced search for dark matter axions. *Nature*. **590**, 238–242 (2021).

14. G. A. Oakes, V. N. Ciriano-Tejel, D. F. Wise, M. A. Fogarty, T. Lundberg, C. Lainé, S. Schaal, F. Martins, D. J. Ibberson, L. Hutin, B. Bertrand, N. Stelmashenko, J. W. A. Robinson, L. Ibberson, A. Hashim, I. Siddiqi, A. Lee, M. Vinet, C. G. Smith, J. J. L. Morton, M. F. Gonzalez-Zalba, Fast high-fidelity single-shot readout of spins in silicon using a single-electron box. *Phys. Rev. X*. **13**, 011023 (2023).

15. A. Bienfait, J. J. Pla, Y. Kubo, M. Stern, X. Zhou, C. C. Lo, C. D. Weis, T. Schenkel, M. L. W. Thewalt, D. Vion, D. Esteve, B. Julsgaard, K. Mølmer, J. J. L. Morton, P. Bertet, Reaching

the quantum limit of sensitivity in electron spin resonance. *Nat. Nanotechnol.* **11**, 253–257 (2016).

16. C. Eichler, A. J. Sigillito, S. A. Lyon, J. R. Petta Electron spin resonance at the level of 104 spins using low impedance superconducting resonators. *Phys. Rev. Lett.* **118**, 037701 (2017).

17. R. P. Budoyo, K. Kakuyanagi, H. Toida, Y. Matsuzaki, W. J. Munro, H. Yamaguchi, S. Saito, Electron paramagnetic resonance spectroscopy of Er3+:Y2SiO5 using a Josephson bifurcation amplifier: Observation of hyperfine and quadrupole structures. *Phys. Rev. Materials* **2**, 011403 (2018).

18. R. P. Budoyo, K. Kakuyanagi, H. Toida, Y. Matsuzaki, S. Saito, Electron spin resonance with up to 20 spin sensitivity measured using a superconducting flux qubit. *Appl. Phys. Lett.* **116**, 194001 (2020).

19. E. Albertinale, L. Balembois, E. Billaud, V. Ranjan, D. Flanigan, T. Schenkel, D. Estève, D. Vion, P. Bertet, E. Flurin, Detecting spins by their fluorescence with a microwave photon counter. *Nature*. **600**, 434–438 (2021).

20. E. Billaud, L. Balembois, M. L. Dantec, M. Rančić, E. Albertinale, S. Bertaina, T. Chanelière, P. Goldner, D. Estève, D. Vion, P. Bertet, E. Flurin, Microwave fluorescence detection of spin echoes. *arXiv*. 2208.13586 (2022).

21. Z. Wang, L. Balembois, M. Rančić, E. Billaud, M. le Dantec, A. Ferrier, P. Goldner, S. Bertaina, T. Chanelière, D. Esteve, D. Vion, P. Bertet, E. Flurin, Single-electron spin resonance detection by microwave photon counting. *Nature* **619**, 276–281 (2023).

22. N. T. Bronn, Y. Liu, J. B. Hertzberg, A. D. Córcoles, A. A. Houck, J. M. Gambetta, J. M. Chow, Broadband filters for abatement of spontaneous emission in circuit quantum electrodynamics. *Appl. Phys. Lett.* **107**, 172601 (2015).

23. A. T. Asfaw, A. J. Sigillito, A. M. Tyryshkin, T. Schenkel, S. A. Lyon, Multi-frequency spin manipulation using rapidly tunable superconducting coplanarwaveguide microresonators. *Appl. Phys. Lett.* **111**, 032601 (2017).

24. M. R. Vissers, R. P. Erickson, H. S. Ku, L. Vale, X. Wu, G. C. Hilton, D. P. Pappas Low-noise kinetic inductance traveling-wave amplifier using three-wave mixing. *Appl. Phys. Lett.*, **108**, 012601 2016.

25. C. M. Wilson, T. Duty, M. Sandberg, F. Persson, V. Shumeiko, P. Delsing, Photon generation in an electromagnetic cavity with a time-dependent boundary. *Phys. Rev. Lett.*, **105**, 233907 (2010).

26. W. Wustmann, V. Shumeiko, Parametric resonance in tunable superconducting cavities. *Phys. Rev. B,* **87**, 184501 (2013).

27. H. Wang, J. Gao, M. R. Vissers, T. Brecht, A. Dunsworth, D. P. Pappas, J. Mutus, Materials loss measurements using superconducting microwave resonators. *Rev. Sci. Instrum.* **91**, 091101 (2020).

28. A. Bienfait, J. J. Pla, Y. Kubo, X. Zhou, M. Stern, C. C. Lo, C. D. Weis, T. Schenkel, D. Vion, D. Esteve, J. J. L. Morton, P. Bertet Controlling spin relaxation with a cavity. *Nature* **531**, 74–77 (2016).

29. G. Wolfowicz, S. Simmons, A. M. Tyryshkin, R. E. George, H. Riemann, N. V. Abrosimov, P. Becker, H.-J. Pohl, S. A. Lyon, M. L. W. Thewalt, J. J. L. Morton, Decoherence mechanisms of 209Bi donor electron spins in isotopically pure 28Si. *Phys. Rev. B,* **86**, 245301 (2012).

30. V. Ranjan, J. O’Sullivan, E. Albertinale, B. Albanese, T. Chanelière, T. Schenkel, D. Vion, D. Esteve, E. Flurin, J. J. L. Morton, P. Bertet, Multimode storage of quantum microwave fields in electron spins over 100 ms. *Phys. Rev. Lett.* **125** 210505 (2020).

31. J. O'Sullivan *et al.*, Random-access quantum memory using chirped pulse phase encoding. *Phys. Rev. X* **12**, 041014 (2022).

32. J. J. L. Morton, P. Bertet, Storing quantum information in spins and high-sensitivity ESR. *J. Magn. Reson.* **287**, 128–139 (2018).

33. L. J. Berliner, G. R. Eaton, S. S. Eaton, Distance Measurements in Biological Systems by EPR. Springer Science & Business Media, 2006.

34. D. Niepce, J. J. Burnett, M. G. Latorre, J. Bylander, Geometric scaling of two-level-system loss in superconducting resonators. *Supercond. Sci. Technol.*, **33**, 025013 (2020).

35. J. M. Martinis, K. B. Cooper, R. Mc Dermott, M. Steffen, M. Ansmann, K. D. Osborn, K. Cicak, S. Oh, D. P. Pappas, R. W. Simmonds, C. C. Yu, Decoherence in Josephson qubits from dielectric Loss. *Phys. Rev. Lett.*, **95**, 210503 (2005).

36. J. Gao, M. Daal, A. Vayonakis, S. Kumar, J. Zmuidzinas, B. Sadoulet, B. A. Mazin, P. K. Day, H. G. Leduc Experimental evidence for a surface distribution of two-level systems in superconducting lithographed microwave resonators. *Appl. Phys. Lett.*, **92**, 152505 (2008).

37. J. H. Ungerer, D. Sarmah, A. Kononov, J. Ridderbos, R. Haller, L. Y. Cheung, C. Schönenberger, Performance of high impedance resonators in dirty dielectric environments. *EPJ Quantum Technol.* **10**, 41 (2023).

38. P. C. Humphreys, N. Kalb, J. P. J. Morits, R. N. Schouten, R. F. L. Vermeulen, D. J. Twitchen, M. Markham, R. Hanson Deterministic delivery of remote entanglement on a quantum network. *Nature*, **558**, 268–273 (2018).

39. A. Ourjoumtsev, R. Tualle-Brouri, J. Laurat, P. Grangier Generating optical Schrödinger kittens for quantum information processing. *Science* **312**, 83–86 (2006).

40. J. Anders, A. Angerhofer, G. Boero *et al.*, K-band single-chip electron spin resonance detector. *J. Magn. Reson.*, **217**, 19–26 (2012)

41. S. Probst, F. B. Song, P. A. Bushev, A. V. Ustinov, M. Weides, Efficient and robust analysis of complex scattering data under noise in microwave resonators. *Rev. Sci. Instrum.* **86**, 024706 (2015).

42. J. J. Pla, A. Bienfait, G. Pica, J. Mansir, F. A. Mohiyaddin, Z. Zeng, Y. M. Niquet, A. Morello, T. Schenkel, J. J. L. Morton, P. Bertet Strain-induced spin-resonance shifts in silicon devices. *Phys. Rev. Applied*, **9**, 044014 (2018).

43. C. P. Slichter. Princples of Magnetic Resonance. Springer, Berlin, 3rd ed. edition, 1996. 12 927.

44. M. R. Vissers, J. Hubmayr, M. Sandberg, S. Chaudhuri, C. Bockstiegel, J. Gao, Frequency-tunable superconducting resonators via nonlinear kinetic inductance. *Appl. Phys. Lett.* **107**, 062601 (2015).

45. A. Bruno, G. de Lange, S. Asaad, K. L. van der Enden, N. K. Langford, L. DiCarlo, Reducing intrinsic loss in superconducting resonators by surface treatment and deep etching of silicon substrates. *Appl. Phys. Lett.* **106**, 182601 (2015).

46. D. Niepce, J. Burnett, J. Bylander, High kinetic inductance NbN nanowire superinductors. *Phys. Rev. Applied* **11**, 044014 (2019).

47. R. P. Erickson, M. R. Vissers, M. Sandberg, S. R. Jefferts, D. P. Pappas, Frequency comb generation in superconducting resonators. *Phys. Rev. Lett.* **113**, 187002 (2014).

48. M. C. Cassidy, A. Bruno, S. Rubbert, M. Irfan, J. Kammhuber, R. N. Schouten, A. R. Akhmerov, L. P. Kouwenhoven Demonstration of an ac Josephson junction laser. *Science*, **355**, 939–942 (2017).

49. S. Khan, H. E. Türeci Frequency combs in a lumped-element josephson-junction circuit. *Phys. Rev. Lett.*, **120**, 153601 (2018).

50. P. Lu, T.-C. Chien, X. Cao, O. Lanes, C. Zhou, M. J. Hatridge, S. Khan, H. E. Türeci, Nearly quantum-limited Josephson junction frequency-comb synthesizer. *Phys. Rev. Applied*, **15**, 044031 (2021).

51. S. P. Wang, Z. Chen, T. Li, Control lable microwave frequency comb generation in a tunable superconducting coplanar-waveguide resonator. *Chinese Phys. B* **30**, 048501 (2021).

52. J. Y. Qiu, A. Grimsmo, K. Peng, B. Kannan, B. Lienhard, Y. Sung, P. Krantz, V. Bolkhovsky, G. Calusine, D. Kim, A. Melville, B. M. Niedzielski, J. Yoder, M. E. Schwartz, T. P. Orlando, I. Siddiqi, S. Gustavsson, K. P. O'Brien, W. D. Oliver, Broadband squeezed microwaves and amplification with a Josephson travelling-wave parametric amplifier. *Nat. Phys.* **19**, 706–713 (2023).
